# TET2-driven activation of AGO2 links epigenetic remodeling to myeloid commitment and leukemia

**DOI:** 10.1126/sciadv.aee5701

**Published:** 2026-10-23

**Authors:** Aleksey Lazarenkov, Gemma Valcárcel, Alba Martinez, Clara Berenguer, Anna V. López-Rubio, Mireia Obiols, Claudia Fontanet, Ana Barba, Juan José Rodríguez-Sevilla, Gregoire Stik, Irmela Jeremias, Pablo Menendez, José Luis Sardina

**Affiliations:** ^1^ Epigenetic Control of Haematopoiesis Group, Josep Carreras Leukaemia Research Institute (IJC), Campus Can Ruti, Badalona, Spain.; ^2^ Stem Cell Biology, Developmental Leukemia and Immunotherapy Group, Josep Carreras Leukaemia Research Institute (IJC), Campus Hospital Clínic, Barcelona, Spain.; ^3^ Red de Terapias Avanzadas (TERAV), Instituto de Salud Carlos III, Madrid, Spain.; ^4^ Endocrine Regulatory Genomics, Department of Experimental & Health Sciences, University Pompeu Fabra, 08003 Barcelona, Spain.; ^5^ Department of Hematology, Hospital Clinic Barcelona, Barcelona, Spain.; ^6^ Institut d’Investigacions Biomèdiques August Pi i Sunyer (IDIBAPS), Barcelona, Spain.; ^7^ Nuclear Architecture in Leukemia Group, Josep Carreras Leukaemia Research Institute (IJC), Campus Can Ruti, Badalona, Spain.; ^8^ Research Unit Apoptosis in Hematopoietic Stem Cells, Helmholtz Munich, German Research Center for Environmental Health (HMGU), Munich, Germany.; ^9^ German Cancer Consortium (DKTK), partner site Munich, a partnership between DKFZ and University Hospital LMU Munich, Munich, Germany.; ^10^ Pediatric Cancer Centre Barcelona-Institut de Recerca Sant Joan de Deu (PCCB-SJD), Barcelona, Spain.; ^11^ Department of Biomedicine, University of Barcelona, Barcelona, Spain.; ^12^ Centro de Investigación Biomédica en Red de Cáncer (CIBERONC), Madrid, Spain.; ^13^ Institució Catalana de Recerca i Estudis Avançats (ICREA), Barcelona, Spain.

## Abstract

DNA methylation dynamics shape hematopoietic differentiation and leukemogenesis, yet how the dioxygenase TET2—frequently mutated in myeloid malignancies—directs lineage-specific regulatory programs remains unclear. Here, we integrated DNA methylation, chromatin accessibility, three-dimensional (3D) genome architecture, transcriptional profiling, and TET2 chromatin occupancy to define TET2-dependent control of human myeloid commitment. We found that TET2-bound regulatory regions gain short- and long-range chromatin interactions and that a distinct subset of distal, enhancer-enriched sites undergoes TET2-driven demethylation and activation. Among these, we identified and functionally validated an *AGO2* myeloid-specific intragenic enhancer that is frequently hypermethylated in patients with *TET2*-mutant acute myeloid leukemia. *AGO2* expression stratifies patient survival, and AGO2 depletion abrogates leukemic engraftment in vivo. These findings uncover a TET2-AGO2 regulatory axis that integrates epigenetic remodeling, 3D genome reorganization, and leukemic fitness, and they highlight AGO2 as a potential biomarker and therapeutic target in myeloid leukemia.

## INTRODUCTION

DNA methylation (DNAm) at CpG dinucleotides is the main epigenetic modification in mammalian genomes and plays essential roles in development, differentiation, and cancer (*1*, *2*). Although traditionally linked to transcriptional silencing, DNAm gain at certain promoters has been observed to coincide with increased gene expression, highlighting its context-dependent regulatory role (*3*). DNAm can be removed passively through the progressive dilution of methylation marks during DNA replication or actively via oxidation reactions catalyzed by the Ten-eleven-translocation (TET) family of dioxygenases (*4*). In the active pathway, TET enzymes iteratively oxidize 5-methylcytosine (5mC) to 5-hydroxymethylcytosine (5hmC) and subsequent oxidized derivatives, which are then either lost through replication or enzymatically excised, ultimately restoring unmodified cytosine (*4*).

During blood cell fate decisions, the transcriptional impact of DNAm is shaped by the methylation sensitivity of lineage-instructive transcription factors (TFs). For example, Myc, Myb, and other basic helix-loop-helix TFs show reduced binding to methylated DNA, whereas Kruppel-like factors (KLFs), such as KLF4, retain or even enhance binding in the presence of 5mC (*5*). Notably, KLF4, CEBPA, and other pioneer myeloid TFs can recruit TET2 to CREs, linking methylation-insensitive TF binding to targeted demethylation during myeloid commitment (*6*, *7*). These observations therefore underscore the essential role of TET2-mediated enzymatic activity in modulating transcriptional outputs. *TET2* is broadly expressed throughout hematopoiesis and is one of the most recurrently mutated genes in myeloid malignancies, including acute myeloid leukemia (AML), myeloproliferative neoplasms, and myelodysplastic syndromes (*8*–*10*), as well as in clonal hematopoiesis (*11*, *12*). Its loss of function drives differentiation arrest, myeloid bias, and aberrant inflammatory gene expression (*13*–*17*), yet the direct molecular mechanisms underlying these phenotypes remain poorly defined, mainly due to long-standing technical challenges in accurately mapping TET2 chromatin occupancy during hematopoietic differentiation.

To address this gap, we performed an integrative multiomics analysis centered on TET2 binding during human myeloid commitment. Data revealed that TET2-bound regions undergo strengthening of both short- and long-range chromatin self-interactions during lineage specification, suggesting a role in higher-order genome organization. Notably, we also identified a discrete, enhancer-enriched subset of TET2 sites that become demethylated and chromatin activated specifically during myeloid commitment, comprising 16 high-confidence targets. Among these, *AGO2* displayed the strongest methylation-expression response to TET2 depletion, driven by an intragenic enhancer that is selectively demethylated in the myeloid lineage and functionally regulates *AGO2* expression. Perturbation of this TET2-AGO2 axis accelerated myeloid differentiation, altered inflammatory and proliferative programs, and profoundly impaired leukemic development in vivo, while *AGO2* expression and methylation status were associated with distinct clinical features and outcomes in AML.

## RESULTS

### A Flag-tag CRISPR knockin strategy enables robust mapping of TET2 occupancy during myeloid commitment

Despite being a well-established facilitator of myeloid gene activation (*6*, *13*, *15*, *18*, *19*), the cis-regulatory elements (CREs) through which TET2 exerts this function remain poorly defined, largely due to technical challenges in mapping its precise genomic distribution. While TET2 occupancy has been characterized in terminally differentiated human myeloid cells (*20*), its function during myeloid lineage commitment remains largely unexplored. To address this critical gap, we used a CEBPA-driven myeloid commitment model that efficiently converts a B cell leukemia line into functionally induced macrophages (iMacs) (*21*). These iMacs recapitulate key features of primary macrophages, including robust phagocytic capacity, inflammasome competence, and responsiveness to inflammatory and pathogenic stimuli (*21*–*24*). To overcome the technical limitations to profile TET2, we introduced a Flag-tag at the *TET2* locus using CRISPR knockin, generating tagged B cells that were subsequently induced toward the myeloid fate (Fig. 1A). TET2 genomic occupancy was then profiled at the onset of the myeloid commitment [72 hours postinduction (hpi)] in biological duplicates using a double-cross-linking protocol and anti-Flag immunoprecipitation followed by high-throughput sequencing (Fig. 1A). Notably, at 72 hpi, TET2 is increased at both the mRNA and protein levels (fig. S1A), coinciding with activation of the myeloid gene program (fig. S1B).

**Fig. 1.**
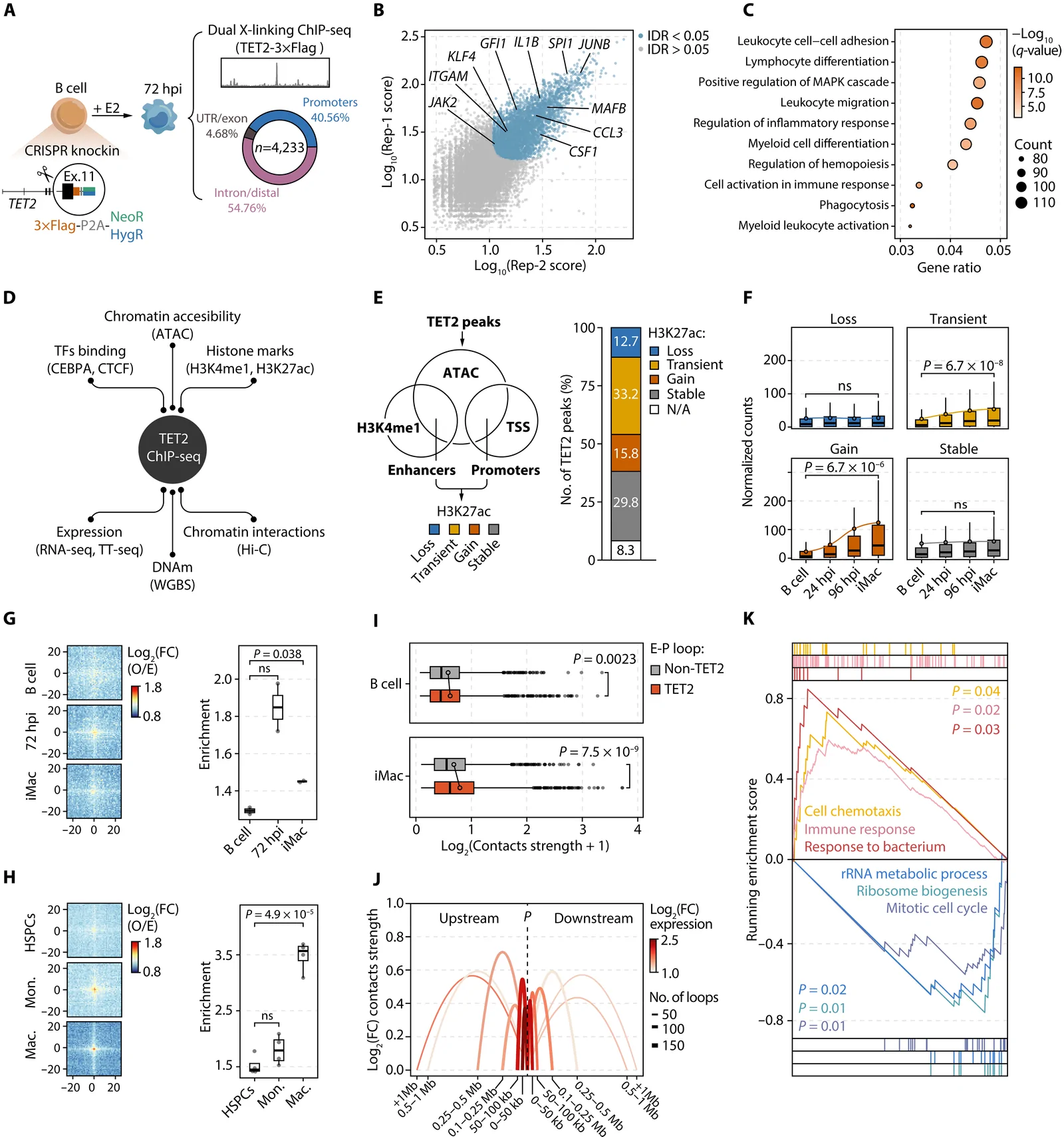
TET2 occupancy links E-P architecture to activation of myeloid gene programs. (**A**) Schematic overview of the strategy used for TET2-3xFlag tagging and chromatin immunoprecipitation. The number of significant peaks and their annotation are shown. (**B**) Scatterplot of the IDR analysis showing common TET2 binding sites between the two biological replicates. (**C**) GO enrichment analysis of genes associated with TET2 binding sites. Selected biological processes from the top 20 most significant GO terms are shown. (**D**) Schematic overview of TET2 genomic distribution data integrated with available datasets in the myeloid commitment cellular model (*23*, *24*, *26*). (**E**) Left: Schematic overview of the strategy used to classify TET2 binding sites into CREs. Right: Distribution of TET2-bound CREs according to their activation kinetics (H3K27ac levels). N/A, not available. (**F**) Gene expression (RNA-seq) dynamics during myeloid commitment for genes associated with the TET2-bound CREs. Dots with a stroke represent average values. ns, not significant. (**G**) Left: APA plots showing long-range interactions (5 to 10 Mb) between TET2-bound regions during myeloid commitment. O/E: observed over expected. Right: APA quantification showing the enrichment of the central bin (iMac versus B cell, *n* = 2 per time point). (**H**) Left: APA plots showing long-range interactions (5 to 10 Mb) between TET2-bound regions during primary healthy myelopoiesis. Right: APA quantification showing the enrichment of the central bin [macrophages (Mac.) versus HSPCs, *n* = 4 per time point]. Mon., monocytes. (**I**) Comparison of contact strength between TET2- and non–TET2-bound E-P sites during myeloid commitment. (**J**) Arc diagram depicting average contact strength and differential expression (iMac versus B cell) of genes linked to TET2-bound E-P sites during myeloid commitment. (**K**) Gene set enrichment analysis (GSEA) of genes associated with TET2-bound E-P sites (iMac versus B cell). Selected significantly enriched terms are plotted.

Our chromatin occupancy analysis identified 4233 highly confident TET2 binding sites [irreproducibility discovery rate (IDR) <0.05], including regions associated with key myeloid regulators such as *SPI1*, *KLF4*, *MAFB*, or *GFI1* (Fig. 1, A and B). In line with this, Gene Ontology (GO) analysis revealed that TET2-bound regions were enriched for genes related to myeloid commitment and macrophage functions (Fig. 1C). Notably, most TET2 peaks localized to promoters and distal intra/intergenic elements (95.3%) (Fig. 1A and fig. S1C) and were positioned either within 1 kb of the TSS (transcription start site; 29.2%) or at typical enhancer distances (10 to 100 kb; 34.1%) from their nearest gene (fig. S1D) (*25*), consistent with a role of TET2 occupancy in transcriptional regulation.

To refine the characterization of TET2 genomic distribution, we integrated multiomics datasets capturing chromatin, transcriptional, and DNAm dynamics during myeloid commitment (Fig. 1D) (*23*, *24*, *26*). We classified TET2 binding sites as promoters or enhancers on the basis of chromatin accessibility and H3K4me1 enrichment and assessed their activation by H3K27ac dynamics during myeloid commitment (Fig. 1E, left). Most TET2-bound regions (91.7%) overlapped with CREs, particularly those showing stable or transient H3K27ac gain during commitment (Fig. 1E, right, and fig. S1E). Notably, these changes correlated with gene up-regulation (Fig. 1F), highlighting TET2’s role in promoting myeloid gene expression. Together, these results reveal extensive chromatin reconfiguration at TET2-bound regions during myeloid commitment, hinting at a coordinated reorganization of the underlying genomic landscape.

### TET2 binding sites undergo three-dimensional chromatin reorganization during myeloid commitment

To gain insight into chromatin dynamics of TET2-bound CREs during myeloid commitment, we integrated our occupancy data with genome-wide chromatin interaction maps (Hi-C) (Fig. 1D) (*23*). Motivated by a recent report suggesting that TET2 forms biomolecular condensates (*27*), we investigated whether its binding is associated with changes in three-dimensional (3D) chromatin architecture. We observed a clear enrichment of long-range interactions (5 to 10 Mb) between TET2-bound sites during myeloid commitment (Fig. 1G). Notably, a similar pattern was detected during primary hematopoietic stem and progenitor cell (HSPC)–to–macrophage differentiation (Fig. 1H), suggesting a potential role for TET2 in coordinating 3D chromatin rewiring. CEBPA, which highly colocalizes (fig. S1F) and interacts with TET2 (*6*), has also been reported to drive the formation of active 3D hubs through phase separation (*28*), suggesting a potential cooperative function of both proteins during myeloid commitment. Conversely, these TET2-associated 3D chromatin changes seem to occur independently of CCCTC-binding factor (CTCF), as no changes in its occupancy were observed at TET2-bound sites during myeloid commitment (fig. S1G).

While long-range contacts reflect higher-order chromatin organization, enhancer-promoter (E-P) loops directly link distal regulatory elements to gene transcription. To explore how TET2 contributes to local gene regulation, we analyzed TET2-bound E-P interactions. Using our previous E-P definition (Fig. 1E), we identified 1358 loops occupied at both ends by TET2, which exhibited elevated contact frequency during myeloid commitment (fig. S1H). Notably, TET2-bound E-P loops were significantly stronger than random pairs in iMacs (Fig. 1I), indicating that TET2 binding may promote E-P association for transcriptional activation.

These loops were enriched in short-range contacts (<250 kb) that correlated with gene up-regulation during myeloid commitment (Fig. 1J). The associated transcriptional programs reflected activation of immune-related genes and repression of cell cycle regulators, consistent with myeloid lineage acquisition (Fig. 1K). To gain mechanistic insight, we next investigated whether TET2 catalytic activity contributes to loop establishment. We quantified DNAm levels from whole-genome bisulfite sequencing (WGBS) data (Fig. 1D) at TET2 peaks within the E-P subset. Overall, DNAm changes did not significantly correlate with strengthened interactions during myeloid commitment (fig. S1I). Nonetheless, specific promoters and, to a greater extent, enhancers exhibited both demethylation and increased contact strength, as observed at key immune-related genes such as *RUNX3*, *IL3RA*, *IFNGR2*, or *NOD2* (fig. S1J). Overall, while DNA demethylation seems insufficient to drive global E-P loop formation, locus-specific TET2 binding and associated demethylation may facilitate 3D chromatin contacts that enable transcriptional activation of myeloid lineage–specific genes.

### An enhancer-enriched subset of TET2 binding sites is specifically demethylated and activated during myeloid commitment

We next examined DNAm dynamics across all TET2-bound regions to assess their impact on chromatin and transcriptional activity during myeloid commitment. Quantitative analysis revealed methylome remodeling from 24 hpi onward (fig. S2A), coinciding with TET2 protein accumulation at 72 hpi (fig. S1A). Notably, only ∼6% of the total DNAm signal was lost between B cell and iMac stages (fig. S2A), indicating that, despite widespread TET2 occupancy, demethylation is likely confined to a selective subset of CREs. To address this, we classified all TET2-binding sites as either TET2-bound methylation-stable peaks (T2-MSPs) or TET2-bound methylation-dynamic peaks (T2-MDPs) using a ΔDNAm >0.15 threshold between the iMac and B cell stages (Fig. 2, A and B). The distribution of the two groups was uneven: T2-MSPs, showing an average DNAm loss of only 1.1%, accounted for 84.2% of all sites (3427 regions), whereas T2-MDPs, which lost >35% of DNAm, represented just 15.8% (642 regions) (Fig. 2, A and B). Consistently, T2-MDPs exhibited significantly greater DNAm loss across all time points of myeloid commitment (fig. S2B), suggesting them as a dynamically remodeled subset of TET2-bound regulatory elements.

**Fig. 2.**
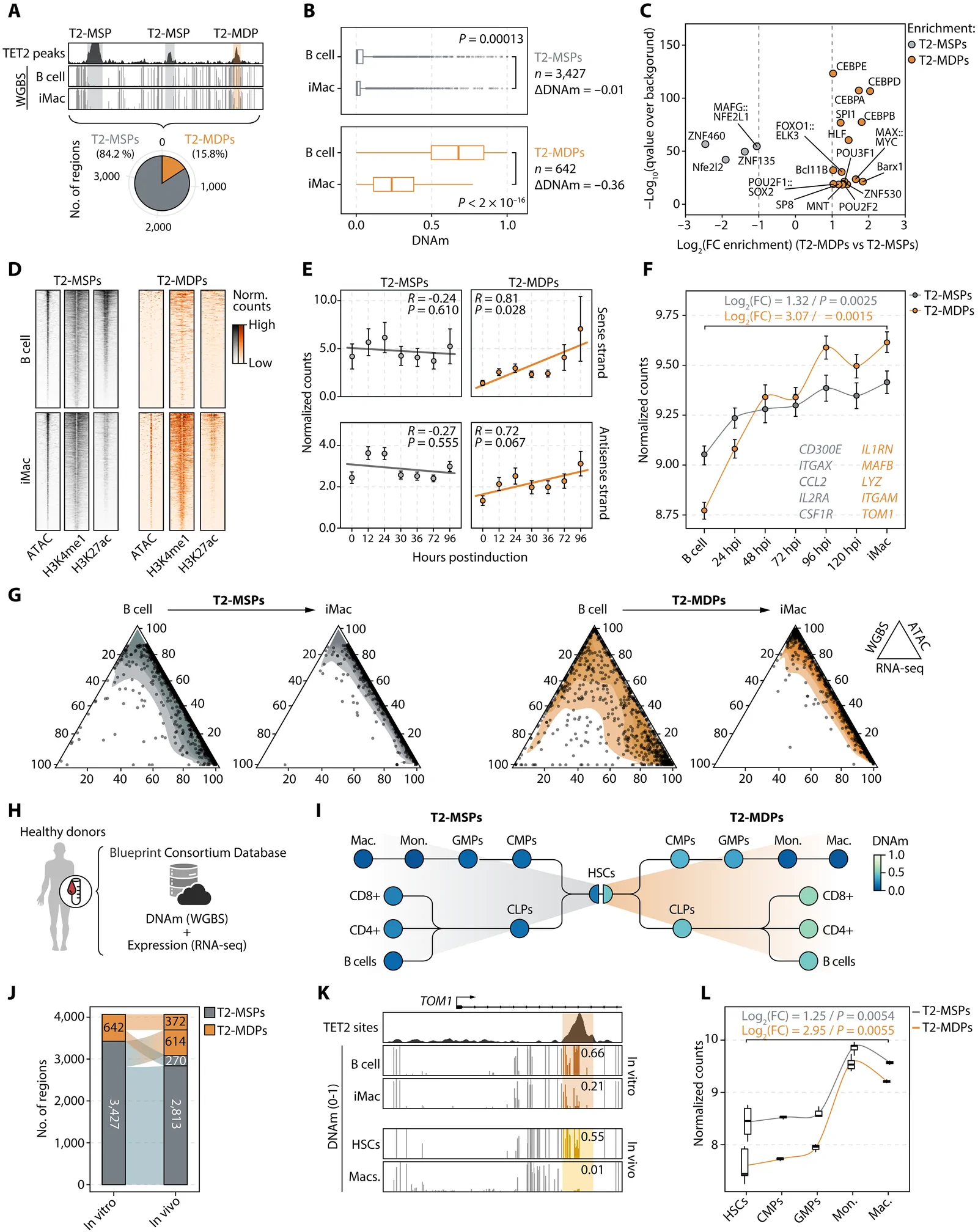
TET2 binding leads to DNA demethylation and activation of a highly dynamic subset of CREs during myeloid commitment. (**A**) Top: Schematic overview of the strategy used for T2-MSPs and T2-MDPs classification. Bottom: Pie chart showing the proportion of T2-MSPs/MDPs. (**B**) DNAm changes (iMac versus B cell) at T2-MSPs and T2-MDPs. (**C**) TF enrichment analysis between T2-MSPs/MDPs. (**D**) Genomic heatmaps showing chromatin accessibility and histone marks (H3K4me1 and H3K27ac) during myeloid commitment at T2-MSPs/MDPs. Signals are shown within a ±5-kb window. (**E**) Nascent RNA (TT-seq) dynamics at T2-MSPs/MDPs during myeloid commitment. The means ± SD are shown (*n* = 2 per time point). Linear regression lines and Pearson correlation *P* values are shown. (**F**) Gene expression (RNA-seq) dynamics during myeloid commitment for T2-MSPs/MDPs–associated genes. The means ± SD are shown. Representative genes are indicated. (**G**) Ternary plots showing changes in gene expression (RNA-seq), DNAm (WGBS), and chromatin accessibility (ATAC-seq) at T2-MSPs/MDPs during myeloid commitment. Shaded regions depict point density. (**H**) Schematic overview of the human datasets used for T2-MSPs/MDPs validation in primary hematopoiesis. (**I**) Bubble plot showing DNAm levels at T2-MSPs/MDPs in primary samples during healthy hematopoiesis (HSCs: *n* = 9; CLPs: *n* = 3; CD8+: *n* = 3; CD4+: *n* = 3; B cells: *n* = 3; CMPs: *n* = 3; GMPs: *n* = 3; Mon.: *n* = 9; Mac.: *n* = 6). (**J**) Comparison of in vitro–defined T2-MSPs/MDPs versus primary HSC-to-macrophage differentiation (in vivo). Shaded regions indicate reshuffling of the proportions between the groups. (**K**) Genome browser snapshot showing a T2-MDP shared between the in vitro and in vivo systems at the TOM1 locus. The shaded region highlights TET2 binding. Average DNAm within the region is shown. (**L**) Gene expression (RNA-seq) dynamics of T2-MSPs/MDPs–associated genes during primary in vivo myeloid commitment. The solid line represents the average values.

To shed light on the distinct DNAm behaviors at TET2-bound regions, we next examined mechanistic differences between the two categories. TET2 binding strength was similar at T2-MSPs and T2-MDPs (fig. S2C). T2-MSPs were enriched at CpG-dense promoter regions, whereas T2-MDPs were biased toward distal sites (10 to 100 kb from genes) and predominantly classified as enhancers (fig. S2, D and E). Next, we performed TF motif enrichment analysis, revealing that T2-MDPs were specifically enriched for key myeloid transcriptional regulators such as SPI1 and the CEBP family (Fig. 2C), including the known TET2 interactor CEBPA (*6*). Consistently, CEBPA occupancy was higher at T2-MDPs during myeloid commitment (fig. S2F), suggesting that it may guide TET2 recruitment and demethylation at these sites. Regarding chromatin and transcriptional dynamics, only T2-MDPs exhibited chromatin activation (measured by ATAC, H3K4me1, and H3K27ac) (Fig. 2D and fig. S2G) and gained bidirectional nascent transcription [by transient transcriptome sequencing (TT-seq)] during myeloid fate acquisition (Fig. 2E), indicating full activation. In contrast, T2-MSPs represent CREs already active in B cells (Fig. 2, D and E, and fig. S2G). Accordingly, although genes associated with both TET2-bound CRE subsets were up-regulated during myeloid commitment, activation was stronger for T2-MDP–linked genes, which started from lower basal levels and reached higher expression than T2-MSP–associated genes {log_2_(fold change) [log_2_(FC)] = 3.07 versus 1.32; respectively} (Fig. 2F).

Overall, T2-MSPs appear to correspond to promoter-proximal, poised elements that sustain basal myeloid identity, while T2-MDPs are likely to characterize latent enhancers of inducible inflammatory programs [STAT1 (signal transducer and activator of transcription 1)/NF-κB (nuclear factor κB)–driven] (fig. S2H). T2-MDPs may thus represent genuine TET2-regulated CREs that undergo demethylation, increase accessibility, and promote strong transcriptional activation (Fig. 2G), offering a framework to evaluate their physiological importance in primary human myeloid differentiation.

### Enhancer-enriched TET2-bound regions show conserved demethylation and activation in primary human myeloid differentiation

To assess the physiological relevance of our findings, we analyzed DNAm dynamics at TET2-bound T2-MSPs and T2-MDPs across primary hematopoietic populations (Fig. 2H). T2-MDPs showed pronounced, lineage-specific demethylation in myeloid cells (monocytes and macrophages) compared with hematopoietic stem cells (HSCs) while remaining highly methylated in lymphoid populations. In contrast, T2-MSPs were broadly unmethylated across all blood lineages (Fig. 2I). Notably, DNAm trajectories at T2-MDPs converged between in vitro and in vivo myeloid commitment despite distinct starting states (fig. S2I), indicating that our model faithfully recapitulates the myeloid-restricted demethylation of a TET2-regulated enhancer–enriched subset.

While these findings already underscore the physiological relevance of our T2-MSPs/T2-MDPs dataset, we next sought to extend our characterization by comparing DNAm dynamics during in vitro and in vivo myeloid commitment. Applying the same ΔDNAm >0.15 threshold used for the in vitro classification revealed some system-specific differences; however, more than half of the in vitro T2-MDPs (372 of 642) were conserved in the in vivo setting (Fig. 2J). These shared regions included CREs of myeloid-related genes such as the *TOM1* intragenic enhancer, which lost 45 and 54% of DNAm during in vitro and in vivo differentiation, respectively (Fig. 2K). Similar demethylation patterns were observed at the *IL1RN* promoter and at a proximal *ITGAM* enhancer (fig. S2J). Consistently, transcriptional profiling of the in vivo T2-MDP–associated genes mirrored the in vitro dynamics, with low basal expression in HSCs followed by marked up-regulation in monocytes and macrophages compared to the T2-MSP–linked genes (Fig. 2L). These changes paralleled the pronounced DNAm loss (Fig. 2I) and the induction of *TET2* expression during primary myeloid differentiation (fig. S2K).

In summary, our comparative analysis of DNAm and transcriptional dynamics during in vitro and primary myeloid commitment reveals a conserved subset of TET2-bound, enhancer-enriched regions that undergo lineage-specific demethylation and robust gene activation, supporting their functional relevance and TET2’s potential role in orchestrating enhancer-driven myeloid gene programs.

### TET2 depletion disrupts lineage-specific DNAm dynamics during myeloid commitment

Having established that a subset of TET2-bound enhancers undergoes lineage-specific demethylation and activation during myeloid commitment, we next investigated to what extent these transcriptional changes depend on TET2’s catalytic activity. To directly assess TET2’s role in gene regulation during myeloid commitment, we generated three doxycycline (Dox)–inducible *TET2* knockdown (TET2 iKD) models (Fig. 3A and fig. S3A). All iKDs exhibited a significant reduction in *TET2* expression (∼50%) following Dox treatment, with shTET2.1 showing the strongest knockdown efficiency (fig. S3A). Notably, shTET2.1 targets exon 3 of *TET2*—a region frequently mutated in myeloid malignancies (*13*, *29*, *30*)—and was therefore selected for downstream characterization (hereafter referred to as shTET2). To ensure sustained *TET2* silencing under Dox treatment, we validated shTET2 efficacy throughout myeloid commitment (fig. S3B). Consistently, shTET2 cells showed markedly lower 5hmC levels (fig. S3C), confirming reduced TET2 catalytic activity. With the shTET2 model validated, we next examined how TET2 depletion affects gene expression and DNAm during myeloid commitment (Fig. 3A). DNAm analyses revealed progressive hypermethylation in shTET2 cells relative to shCTRL cells during myeloid commitment (Fig. 3B and fig. S3D), culminating at the iMac stage with 1794 differentially hypermethylated positions and an average DNAm increase of 21.3% (fig. S3D).

**Fig. 3.**
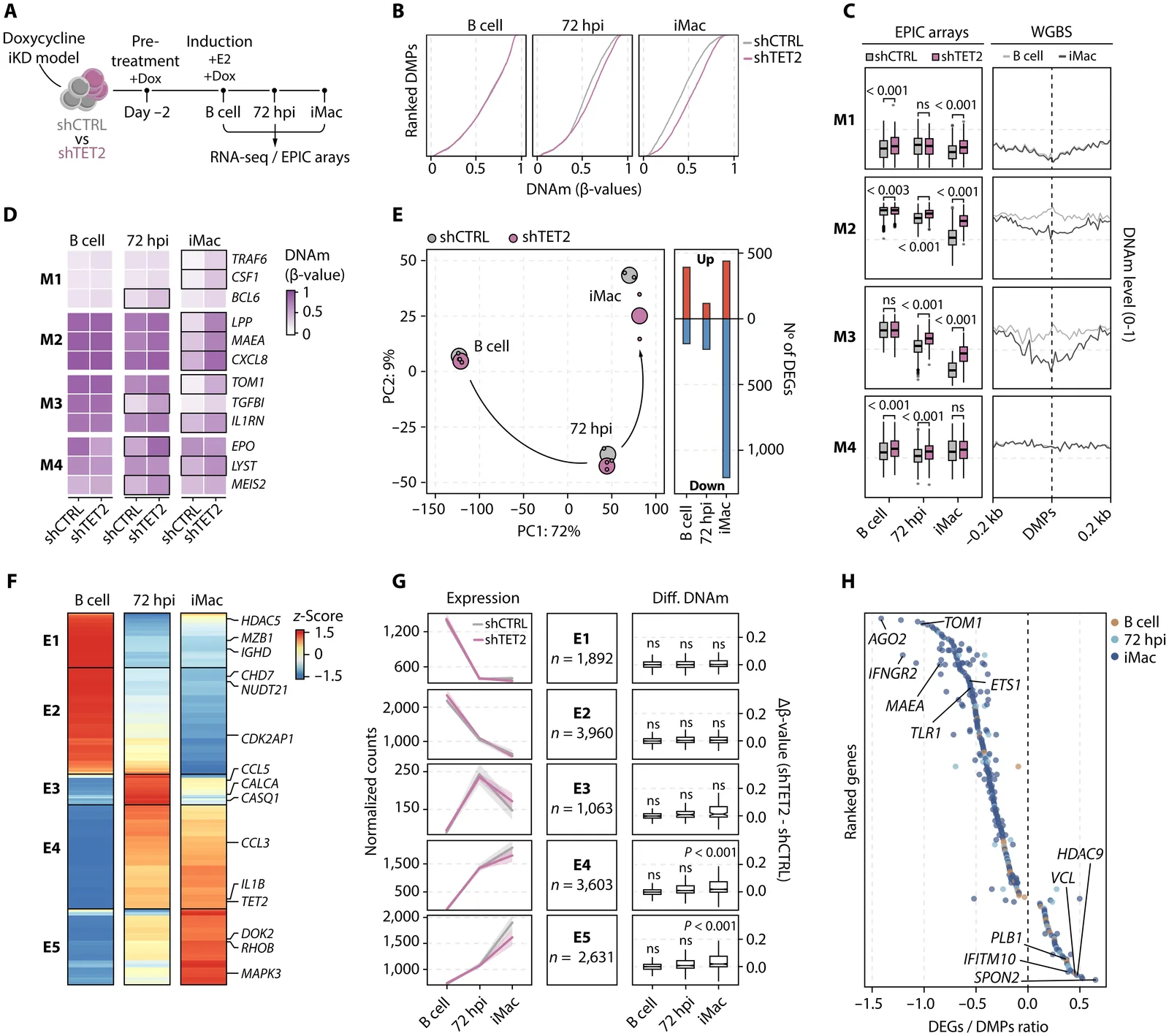
TET2 depletion impairs DNAm-mediated activation of myeloid gene programs. (**A**) Experimental overview of the strategy used to assess the impact of TET2 depletion on DNAm and gene expression during myeloid commitment. (**B**) Ranked DMPs between shTET2 and shCTRL cells during myeloid commitment. (**C**) Left: Quantification of DMPs within DNAm clusters (M1 to M4) during myeloid commitment. Right: Average DNAm profiles (WGBS) centered on DMPs from each cluster (M1 to M4). (**D**) Heatmap of representative hypermethylated DMPs from each DNAm cluster (M1 to M4) during myeloid commitment, with associated gene names indicated. Significant changes (*P* < 0.01) are indicated by a black outline. (**E**) Left: PCA of shTET2 and shCTRL transcriptomes during myeloid commitment. Small dots represent biological replicates (*n* = 2), and large dots represent the average at each time point. Arrows indicate inferred trajectories. Right: Number of up- and down-regulated DEGs in shTET2 cells at each time point. (**F**) Heatmap showing the clustering of transcriptional dynamics (E1 to E5) during myeloid commitment in control conditions [absolute log_2_(FC) > 1]. (**G**) DNAm-expression quantification in expression clusters (E1 to E5) during shTET2 versus shCTRL myeloid commitment. Left: Gene expression in each cluster. Solid lines represent the means, and shaded regions represent the SD (*n* = 2 per time point). Right: DNAm changes associated with genes in each cluster. Only probes within DNAm-dynamic regions (*n* = 11,860), as defined by Valcárcel *et al.* (*24*), were included. (**H**) Ranked ratio of overlapping DEGs and DMPs identified in shTET2 versus shCTRL cells during myeloid commitment. Selected genes with significant hypermethylation and down-regulation are highlighted on the left side of the dashed line, while those with hypermethylation and up-regulation are shown on the right.

To gain a deeper understanding of how TET2 depletion influences DNAm dynamics during myeloid commitment, we clustered all differentially methylated positions (DMPs) into four groups exhibiting distinct temporal trends (fig. S3E). Only DMPs within the M2 and M3 clusters displayed DNAm loss during the process (Fig. 3C, left). These loci overlapped with broader regions [±200 base pairs (bp)] undergoing demethylation during myeloid commitment (Fig. 3C, right), suggesting that the M2 and M3 clusters reflect coordinated regulatory domains rather than isolated DNAm events. Consistent with TET2’s catalytic role, shTET2 cells showed pronounced hypermethylation within M2-M3 clusters at 72 hpi and at the iMac stage (Fig. 3C, left). Genes associated with these clusters were enriched for immune response and myeloid activation pathways (fig. S3F). Despite these differences, all clusters contained key regulators of macrophage differentiation and function—including *CXCL8*, *IL1RN*, *CSF1*, and *EPO* in M1 through M4, respectively (Fig. 3D).

Together, these findings reveal that TET2 loss disrupts the coordinated demethylation of lineage-associated CREs during myeloid differentiation, particularly at loci involved in immune activation and myeloid cell function, thereby establishing a methylation landscape poised to influence transcriptional output.

### TET2 depletion impairs activation of myeloid-specific gene expression programs through defective DNA demethylation

Building on the observed DNAm changes, we next interrogated transcriptional alterations in TET2 iKD cells by RNA sequencing (RNA-seq). We found no evidence of compensatory transcriptional up-regulation of other TET family members following *TET2* knockdown (fig. S3G). Mirroring the progressive changes in DNAm, most of differentially expressed genes (DEGs) were detected at the iMac stage (Fig. 3E and fig. S3H). Notably, principal components analysis (PCA) indicated an overall delay in the transcriptional dynamics of myeloid differentiation in shTET2 conditions (Fig. 3E, left), accompanied by widespread gene silencing (*n* = 1208 DEGs) at the iMac stage (Fig. 3E, right, and fig. S3H). Among the down-regulated genes were key myeloid regulators such as *MAF*, *IL1B*, and *IRF8* (fig. S3H), collectively associated with phagocytosis, immune response, and differentiation processes (fig. S3I).

To gain deeper insight into TET2’s role in shaping normal transcriptional dynamics during myeloid commitment, we applied a clustering approach to define transcriptionally dynamic gene groups during the process and assessed how these patterns were altered upon TET2 depletion. Five transcriptional clusters (E1 to E5) were identified (Fig. 3F), corresponding to the silencing of B cell–related programs (E1) and DNA replication and chromatin organization pathways (E2), transient activation of calcium and phospholipase signaling genes (E3), and induction of immune and myeloid cell activation, leukocyte migration, and vesicle transport processes (E4 and E5) (Fig. 3F and fig. S3J). Integration of transcriptional and DNAm profiles revealed that, while clusters E1 and E2 showed minimal changes, E3 to E5 were markedly affected by TET2 loss. At the transcriptional level, shTET2 cells displayed impaired up-regulation of E4 and E5 genes and, unexpectedly, higher expression of E3 genes compared to controls (Fig. 3G, left). Consistent with these observations, hypermethylation tendencies were detected within the affected clusters, reaching significance at the iMac stage in E4 and E5 (Fig. 3G, right). This relationship was further supported by analyzing the overlap between TET2-dependent DMPs and DEGs, where promoter or genic hypermethylation was predominantly associated with gene silencing in TET2-depleted conditions (Fig. 3H). Notably, this included genes such as *IFNGR2*, *AGO2*, and *TOM1* (fig. S3K), the latter being associated with a TET2-bound methylation dynamic CRE previously identified during myeloid commitment (Fig. 2K).

To independently validate the transcriptional consequences of TET2 loss identified above, we generated CRISPR-Cas9–mediated *TET2* knockout clones targeting the genomic region encoding the catalytic domain of TET2. This approach yielded multiple independent clones displaying different degrees of TET2 depletion, including one clone with undetectable TET2 protein levels (fig. S3L). Several bona fide TET2 target genes identified through integrative analyses (Fig. 3H), including *IFNGR2*, *TOM1*, and *AGO2*, were consistently down-regulated in TET2-deficient clones compared with parental cells and a CRISPR-derived clone retaining wild-type (WT) TET2 (fig. S3M). In addition, *IL1RN*, another TET2 target (fig. S2J) previously shown to regulate myeloid commitment through its promoter methylation status (*24*), was also significantly down-regulated in the knockout clones (fig. S3M).

Together, these findings demonstrate that TET2 loss disrupts the coordinated DNAm and transcriptional programs underlying myeloid commitment. Thus, integrating TET2 occupancy with TET2-dependent epigenetic and transcriptional changes provides a robust framework for identifying direct TET2 target genes.

### Integrative multiomics analyses reveal AGO2 as a direct TET2 target in myeloid commitment

To identify direct TET2 targets, we intersected T2-MDPs with TET2 iKD DMPs and DEGs (Fig. 4A). This analysis yielded 16 candidate genes, including the interferon-γ receptor *IFNGR2*, the histone deacetylase *HDAC9*, and other genes of interest, such a *TOM1*, *NDRG1*, and *AGO2*. Notably, *AGO2*, a key RNA interference regulator, showed the strongest DNAm-expression dependency in TET2-depleted cells (Fig. 3H), uncovering a previously unrecognized role for TET2 in modulating *AGO2* expression, potentially affecting RNA interference–related processes.

**Fig. 4.**
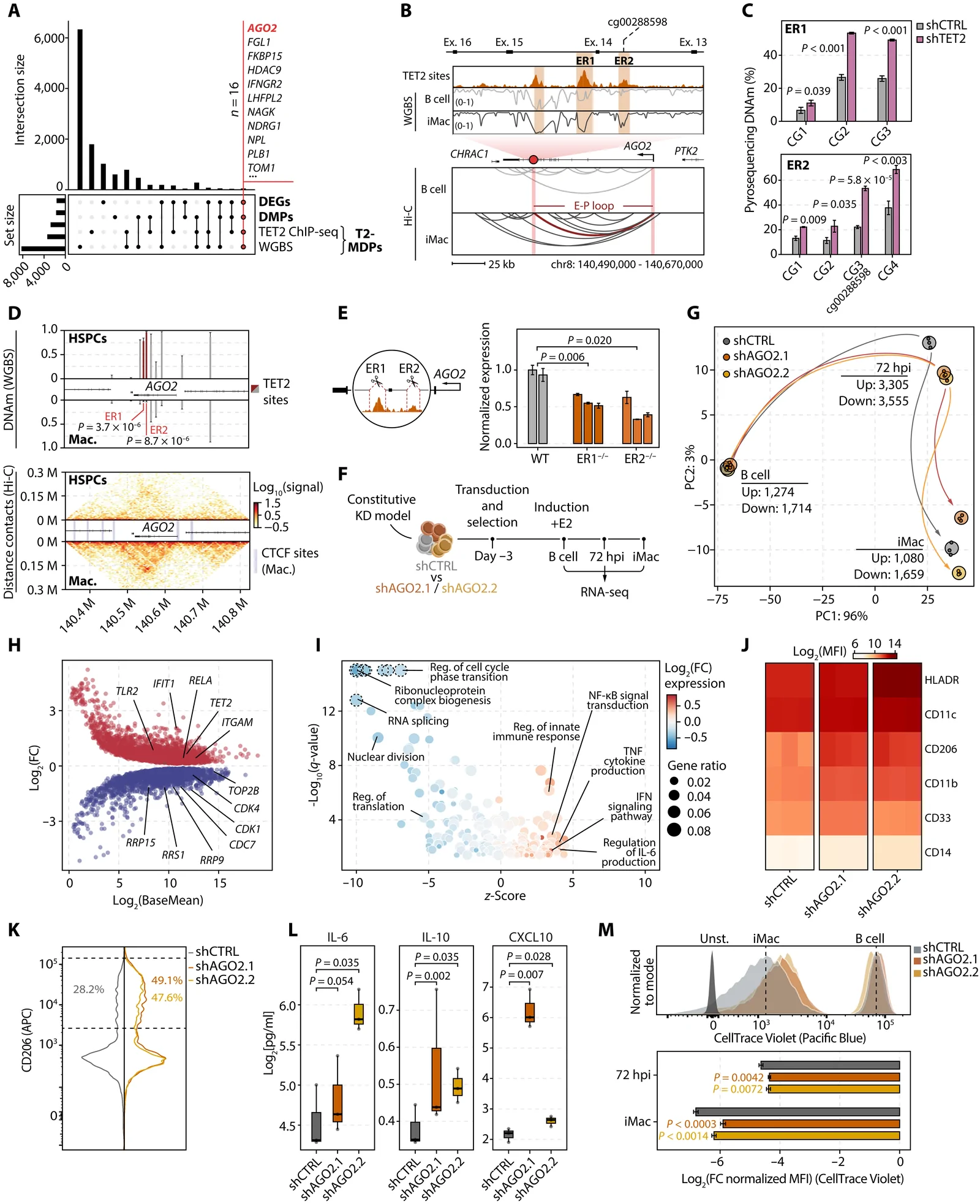
AGO2 is a TET2-dependent regulator of myeloid commitment. (**A**) UpSet plot showing the overlap between T2-MDPs and DMPs/DEGs identified upon TET2 depletion. Candidate are listed in table S1. (**B**) Genome browser snapshot of the *AGO2* ROI. Top: TET2 binding and DNAm dynamics at ER1/ER2. Bottom: Hi-C interaction arc diagram at the *AGO2* locus. Only interactions with a score >1 and a distance >20 kb are shown. The red arc highlights the iMac-specific E-P interaction. (**C**) DNAm levels at ER1/2 in shTET2 and shCTRL iMacs (*n* = 3). (**D**) Genome browser snapshot of the *AGO2* locus during primary myeloid commitment. Top: DNAm at TET2-bound regions (HSPCs: *n* = 4; Mac.: *n* = 4). Bottom: Hi-C contact changes between HSPCs and macrophages. (**E**) Left: Schematic overview of the strategy used to delete the *AGO2* ER1 and ER2 regions. Right: *AGO2* expression in ER1/2^−/−^ versus WT cells (*n* = 3). (**F**) Experimental overview of the strategy used to assess the role of AGO2 during myeloid commitment. KD, knockdown. (**G**) PCA of shAGO2 and shCTRL transcriptomes during myeloid commitment. Small dots indicate replicates (*n* = 3); large dots indicate averages. Arrows indicate inferred trajectories. (**H**) MA plot showing DEGs in shAGO2 versus shCTRL cells at 72 hpi. (**I**) GO enrichment analysis of DEGs in shAGO2 versus shCTRL cells at 72 hpi. The *z*-score indicates over- and underrepresented biological processes. Dotted outlines indicate outlier terms [−log_10_(*q*-value) > 15]. (**J**) Heatmap of myeloid surface marker levels in shAGO2 and shCTRL iMacs. MFI, mean fluorescence intensity. (**K**) Representative CD206 histograms. Percentages of CD206-high cells are shown. (**L**) Cytokine quantification in unstimulated shAGO2 and shCTRL iMacs. (**M**) Proliferation assay in shAGO2 and shCTRL cells during myeloid commitment. Top: Representative CellTrace Violet histograms. Bottom: CellTrace levels normalized to the B cell stage (*n* = 3). Unst., unstained control.

To further explore this regulatory mechanism, we next examined the *AGO2* locus and observed extensive TET2 binding across its gene body, including the intragenic region identified in the multiomics overlap (DMP: cg00288598) (Fig. 4B, top, and fig. S4A). This region displayed features of enhancer activation, marked by the gain of H3K4me1 and H3K27ac during myeloid commitment and CEBPA recruitment from 24 hpi onward (fig. S4A). Chromatin conformation analysis further revealed increased contacts at the *AGO2* locus, including E-P looping between the candidate region and the *AGO2* promoter (Fig. 4B, bottom), consistent with chromatin remodeling and transcriptional changes. Notably, H3K27ac enrichment peaked transiently at 72 hpi, coinciding with expression data (figs. S3K and S4A), suggesting that *AGO2* is transiently up-regulated at intermediate differentiation stages via TET2-dependent DNA demethylation before declining in more committed cells. The region of interest (ROI) encompassed multiple TET2 binding sites that underwent demethylation during myeloid commitment, hereafter referred to as enhancer regions ER1 and ER2 (Fig. 4B, top). The cg00288598 DMP, located within ER2, displayed clear hypermethylation in TET2 iKD cells (fig. S3K). To validate these findings, we quantified DNAm levels at three additional CpGs per site by targeted pyrosequencing, detecting significant ER1/ER2 hypermethylation in iMacs with reduced TET2 levels (Fig. 4C), confirming strong catalytic activity at this locus. Consistent with these observations, the two *TET2* knockout clones displaying the lowest TET2 protein levels (KO3 and KO4; fig. S3L) also showed marked hypermethylation of ER1 and ER2 compared with parental cells and a CRISPR-derived clone retaining WT *TET2* (fig. S4B). Notably, KO3, which lacked detectable TET2 protein, exhibited near-complete methylation at several CpGs within both regions, further supporting a dose-dependent relationship between TET2 levels and DNAm at the ER1 and ER2 regulatory elements (fig. S4B).

We next examined publicly available WGBS and Hi-C datasets from healthy myelopoiesis to assess the physiological relevance of our findings. Consistent with our model, ER1 and ER2 were the only TET2-bound sites exhibiting significant demethylation in macrophages compared with hematopoietic progenitors (Fig. 4D, top). Likewise, chromatin interaction data revealed the macrophage-specific formation of a chromatin domain at the *AGO2* locus, anchored by CTCF sites (Fig. 4D, bottom). Together, these observations supported a physiological role for ER1 and ER2 as *AGO2* regulatory elements during myeloid commitment. To directly assess the regulatory function of these elements, we generated independent CRISPR-Cas9–edited clones harboring deletions of either ER1 or ER2. Deletion of either region resulted in marked *AGO2* down-regulation (Fig. 4E and fig. S4C), demonstrating that both ER1 and ER2 have bona fide enhancer activity and are required for proper *AGO2* expression. Cells lacking either enhancer also displayed increased expression of the myeloid differentiation marker CD11b compared with WT cells (fig. S4D), indicating that perturbation of these regulatory elements is sufficient to alter myeloid cell identity.

Together, these findings identify ER1 and ER2 as functional *AGO2* enhancers that undergo TET2-dependent demethylation during myeloid commitment. The coordinated epigenetic, transcriptional, and chromatin changes observed at this locus across both in vitro and primary hematopoiesis establish *AGO2* as a direct TET2 target, while the phenotypic consequences of ER1 and ER2 disruption suggest that dynamic *AGO2* regulation contributes to the control of myeloid commitment.

### AGO2 restrains myeloid differentiation while supporting inflammatory and proliferative programs

Building on our identification of *AGO2* as a direct TET2 target during myeloid commitment and on the phenotypic consequences of ER1 and ER2 disruption, we sought to investigate its functional role in this process. Notably, AGO2 has previously been implicated in hematopoiesis (*31*, *32*). To this end, we generated two constitutive *AGO2* knockdown cell lines (shAGO2.1 and shAGO2.2), validated efficient depletion, and characterized their behavior upon induction of myeloid commitment (Fig. 4F and fig. S4E). *AGO2* knockdown accelerated myeloid cell fate acquisition, as evidenced by faster loss of the B cell marker CD19 and gain of the myeloid marker CD11b (fig. S4F) and by PCA of the transcriptional landscape (Fig. 4G). Despite minor differences in depletion efficiency (fig. S4E), both shAGO2.1 and shAGO2.2 lines shared more than 60% of DEGs (fig. S4G), showing common down-regulation of metabolism, translation, and cell cycle programs and up-regulation of inflammation-related pathways, including interferon signaling (fig. S4H). Specifically, ribosome and ribosomal RNA metabolism genes, such as *RRP15*, *RRP9*, and *RRS1*, were consistently down-regulated across all time points, while mitotic genes (*CDK1*, *CDK4*, and *CDC7*) were affected primarily at the iMac and 72-hpi stages (Fig. 4, H and I, and fig. S4, I and J). The up-regulated gene set included a broad range of immune response genes, such as *TLR2*, *IFIT1*, *ITGAM*, and *IL1B* (Fig. 4, H and I, and fig. S4, I and J), the latter being elevated at all time points, highlighting the potential impact of reduced AGO2 levels on myeloid rewiring.

To test this hypothesis, we next characterized the phenotype and inflammatory properties of AGO2-depleted iMacs. Flow cytometric analyses of six established myeloid markers (CD11b, CD11c, CD14, CD33, HLA-DR, and CD206) revealed consistently higher levels in AGO2-depleted iMacs compared with control cells (Fig. 4J and fig. S5A), most notably for CD206 (Fig. 4K and fig. S5A), a mannose receptor involved in endocytic and scavenger functions that is typically expressed by differentiated macrophages (*33*, *34*).These findings further support the notion that AGO2 depletion promotes the acquisition of differentiated myeloid features and suggest that the resulting cells may display altered inflammatory properties. Consistent with this idea, AGO2-depleted iMacs exhibited distinct cytokine secretion profiles under basal conditions, including significant changes in interleukin-10 (IL-10) production and increased release of IL-6 or CXCL10 depending on the *AGO2* short hairpin RNA (shRNA) used (Fig. 4L and fig. S5B), mirroring the transcriptional programs identified in the corresponding AGO2-depleted lines (fig. S4H). In contrast, upon stimulation with lipopolysaccharide (LPS), IL-1β, or tumor necrosis factor–α (TNFα), AGO2-depleted iMacs displayed an attenuated cytokine response compared with control cells, most prominently under LPS treatment, with reduced accumulation of IL-6, CXCL10, TNFα, IL-1β, and IL-10 (fig. S5, B and C). Together, these findings suggest that AGO2 depletion promotes acquisition of a differentiated myeloid phenotype while limiting the magnitude of inflammatory responses to external stimuli.

Last, given the consistent down-regulation of cell cycle and mitotic programs in AGO2-depleted cells (Fig. 4, H and I, and fig. S4, I and J), we investigated whether these transcriptional changes translated into altered proliferative capacity. Proliferation analysis using CellTrace Violet dye dilution revealed that AGO2-depleted cells slowed their cell cycle more rapidly than control cells, evident at 72 hpi and in iMacs (Fig. 4M). Notably, a similar effect was observed even at the uninduced B cell stage, with proliferation markedly reduced as early as 3 days postseeding (fig. S5, D and E).

Together, these findings indicate that AGO2 restrains myeloid differentiation while supporting inflammatory and proliferative programs. The ability of AGO2 depletion to promote myeloid commitment and impair cell expansion, together with the observation that myeloid cancer cell lines exhibited increased dependency on AGO2 at higher *AGO2* expression levels, a relationship not observed in other cancer types (fig. S5F), suggests that aberrant *AGO2* expression may contribute to leukemic maintenance, prompting us to investigate its relevance in AML.

### *AGO2* locus methylation status predicts expression and prognosis in AML

The phenotypic consequences of AGO2 depletion, together with the selective dependency of myeloid cancer cell lines on AGO2 activity, prompted us to investigate the relevance of AGO2 in AML, in line with previous observations hinting at its involvement in the disease (*35*). To investigate this possibility, we analyzed DNAm and expression data from publicly available cohorts of adult patients with AML (Fig. 5A). We first examined the impact of *TET2* mutations on *AGO2* DNAm in AML and analyzed EPIC array data from the TCGA-LAML cohort, comparing *TET2*^MUT^ and *TET2*^WT^ samples (Fig. 5B). Consistent with our observations during myeloid commitment, *TET2*^MUT^ samples exhibited widespread hypermethylation, notably including the same *AGO2* cg00288598 DMP that became hypermethylated upon *TET2* knockdown in the myeloid commitment model (Fig. 5B). Analysis of additional AML mutational subgroups revealed that hypermethylation of this probe was largely restricted to patients harboring *TET2* or *IDH1/2* mutations, whereas *DNMT3A*-mutated samples displayed a trend toward lower methylation levels that did not reach statistical significance (fig. S6A). To extend these findings, we examined DNAm across all significant TET2 binding sites within the *AGO2* locus (ROIs: R1 to R7) (Fig. 5C) using WGBS data from patients with AML harboring common mutations. Unsupervised clustering revealed two groups with hypomethylated (A1) and hypermethylated (A2) profiles (Fig. 5D). Consistent with the mutation-specific methylation patterns observed for cg00288598 (fig. S6A), all *TET2*^MUT^ samples (*n* = 8 of 8) and most *IDH1/2*^MUT^ samples (*n* = 4 of 5) clustered within the hypermethylated A2 group, whereas *DNMT3A*^MUT^ samples (*n* = 6 of 8) were enriched in the hypomethylated A1 group (Fig. 5D), indicating that *AGO2* locus methylation is highly sensitive to perturbations of DNAm homeostasis. Quantification of *AGO2* ROIs during healthy hematopoiesis confirmed that R2 and R3, corresponding to ER1 and ER2 (Fig. 4B), were specifically demethylated during monocyte differentiation (fig. S6, B and C). Notably, the same regions were also the most differentially methylated between the A1 and A2 AML clusters (Fig. 5E), highlighting their potential relevance in both physiological and leukemic myelopoiesis. Furthermore, hypermethylation of the *AGO2* cg00288598 probe (within ER2) was consistently associated with poorer overall survival across both the TCGA-LAML and BeatAML cohorts (fig. S6D), likely reflecting the aggressive nature of immature blasts. Consistent with the proposed regulatory relationship, the hypermethylated A2 cluster exhibited lower *AGO2* expression levels than the hypomethylated A1 cluster (Fig. 5F). Last, *TET2* and *AGO2* expression levels showed a modest positive correlation across samples from patients with AML (Fig. 5G), further supporting the existence of a TET2-AGO2 regulatory relationship in human AML. Together, these findings indicate that *AGO2* expression is linked to both epigenetic state and clinical outcome in AML, prompting us to examine how *AGO2* expression levels relate to leukemic phenotypes and disease progression.

**Fig. 5.**
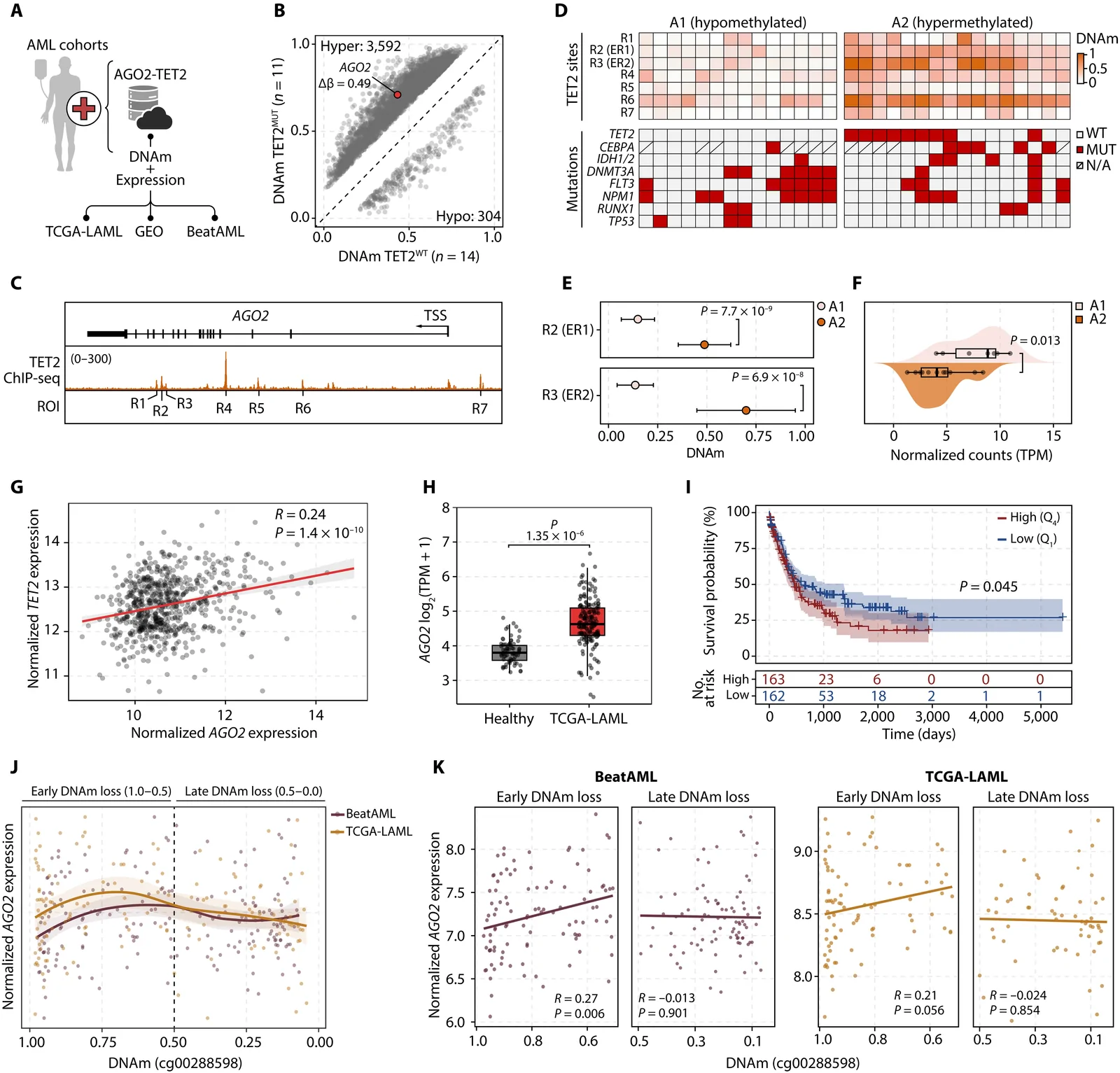
*AGO2* is hypermethylated in patients with *TET2*^MUT^ AML, and its expression is associated with overall survival. (**A**) Schematic overview of the AML expression (RNA-seq) and DNAm (WGBS) datasets analyzed in this study. Publicly available data were obtained from TCGA-LAML, GEO, and BeatAML2. (**B**) Scatterplot showing DMPs between patients with *TET2*^MUT^ and *TET2*^WT^ AML (TCGA-LAML). (**C**) Genome browser snapshot illustrating the AGO2 ROIs (R1 to R7) defined by TET2 binding. (**D**) *AGO2* DNAm status in patients with AML (*n* = 30). Top: Heatmap showing patient clustering (A1 and A2) based on DNAm levels at *AGO2* ROIs (R1 to R7). Bottom: Heatmap showing the mutational profile of each patient. N/A indicates not profiled for the indicated mutation. (**E**) DNAm levels at ER1-ER2 sites across AML clusters (A1 and A2). (**F**) *AGO2* expression in available patient samples in AML clusters A1 (*n* = 7) and A2 (*n* = 11). (**G**) Scatterplot showing the correlation between *AGO2* and *TET2* expression in patients with AML (BEAT-AML2). The linear regression fit is shown as a solid line, and the shaded area denotes the 95% confidence interval. (**H**) *AGO2* expression in patients with AML (TCGA-LAML; *n* = 173) compared with healthy BM samples (GTEx; *n* = 70). Data from GEPIA2 portal (*81*). (**I**) Kaplan-Meier survival plots for patients with high (Q_4_, quartile 4; *n* = 163) versus low (Q_1_, quartile 1; *n* = 162) *AGO2* expression (BEAT-AML2). Shaded areas indicate the 95% confidence interval. (**J**) Scatterplot showing the nonlinear relationship between *AGO2* expression and cg00288598 DNAm from TCGA-LAML (*n* = 145) and BEAT-AML (*n* = 193) cohorts. Expression values were min-max normalized within each dataset. (**K**) Scatterplots showing the correlation between *AGO2* expression and cg00288598 DNAm levels stratified by early (β-values: 1.0 to 0.5) and late (β-values: 0.5 to 0.0) DNAm loss.

### *AGO2* levels associate with AML transcriptional programs and patient outcome

Having established a link between *AGO2* methylation and expression in AML, we next examined the clinical and biological consequences of *AGO2* expression. First, we observed that *AGO2* levels were globally elevated in AML compared with healthy hematopoietic counterparts (Fig. 5H), suggesting that *AGO2* up-regulation may contribute to leukemic biology. Patients with high *AGO2* expression displayed significantly poorer overall survival (Fig. 5I).

To determine whether elevated *AGO2* expression defines distinct biological states, we performed differential expression analysis. This revealed that *AGO2* up-regulation was associated with reduced expression of the proliferation regulators *TP53* and *TP63*, together with increased expression of erythroid-associated regulators such as *TAL1* and *EPOR* (fig. S6, E and F). This transcriptional signature aligns with *AGO2* expression specificity (fig. S6G) and its reported role in murine erythropoiesis (*32*, *34*). Moreover, high-*AGO2* samples were enriched in M6 (acute erythroid) and M7 (acute megakaryoblastic) FAB-AML subtypes (fig. S6H), whereas *NPM1*-mutated AMLs exhibited lower *AGO2* expression than *NPM1*^WT^ cases (fig. S6, I and J). Given the generally favorable prognosis associated with *NPM1* mutations (*36*), this finding further links *AGO2* expression to distinct molecular and clinical features of AML.

Given the previously observed association between *AGO2* methylation and expression, we next asked whether the high- and low-expression groups also differed in *AGO2* DNAm. Notably, a comparison of *AGO2* cg00288598 methylation levels between the high- and low-*AGO2* patient groups revealed no significant differences (fig. S6K). This observation was unexpected, as hypermethylation of the *AGO2* locus was associated with both reduced *AGO2* expression (Fig. 5F) and poorer clinical outcome (fig. S6D), highlighting an apparent discrepancy between the prognostic associations of *AGO2* methylation and *AGO2* expression. To resolve this apparent discrepancy, we examined *AGO2* expression as a function of cg00288598 methylation across the TCGA-LAML and BeatAML cohorts. Although *AGO2* expression generally increased as methylation levels decreased, this relationship was markedly nonlinear (Fig. 5J), with most expression gains occurring during the transition from highly to moderately methylated states and little additional increase below ∼50% methylation (Fig. 5K and fig. S6L). Notably, this pattern mirrors the transient *AGO2* up-regulation observed during myeloid commitment despite progressive DNA demethylation (fig. S3K), suggesting that elevated *AGO2* expression may be associated with leukemic states that retain features of intermediate stages of myeloid differentiation.

Together, these findings suggest that *AGO2* expression defines biologically distinct AML subgroups characterized by specific transcriptional programs, lineage biases, and clinical features. Combined with its association with adverse outcome, these observations raised the possibility that AGO2 may actively contribute to leukemic maintenance, prompting us to test its functional relevance in vivo.

### AGO2 depletion impairs leukemic development across genetically distinct AML-PDX models

Last, to functionally assess whether AGO2 depletion affects leukemogenic capacity in vivo, we evaluated two independent AML patient-derived xenograft (AML-PDX) models derived from patients with distinct clinical and molecular characteristics, including differences in *TET2* mutational status and cytogenetic profile (table S2). AML blasts (AML-579 and AML-602) were transduced with lentiviruses expressing either control shRNA (shCTRL) or *AGO2*-targeting shRNAs (shAGO2.1 and shAGO2.2), resulting in efficient AGO2 protein depletion (fig. S7A). Transduced cells were subsequently injected into recipient mice for longitudinal imaging using the in vivo imaging system (IVIS), survival monitoring, and tissue analyses (Fig. 6A).

**Fig. 6.**
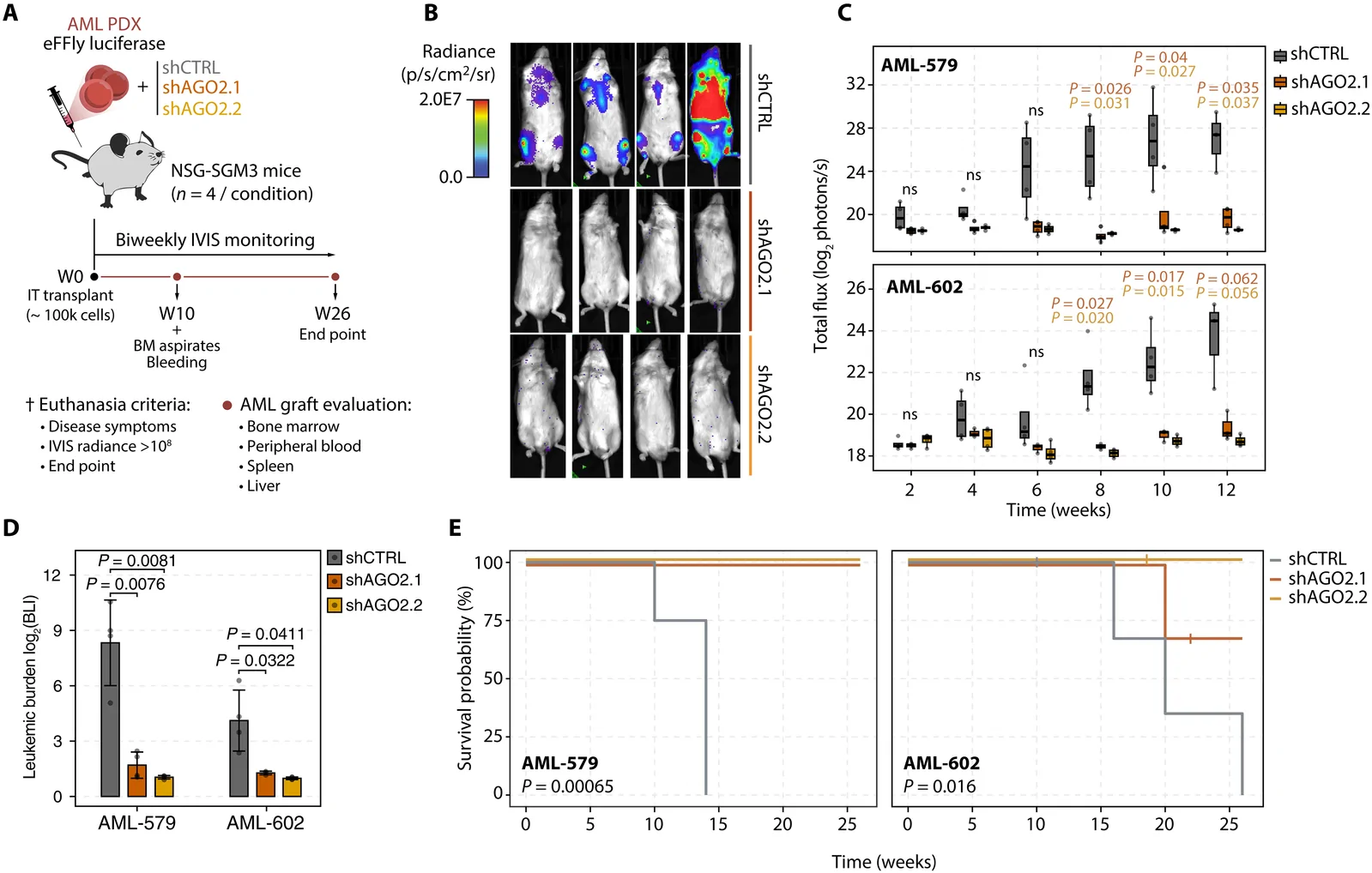
AGO2 depletion impairs in vivo leukemic progression. (**A**) Experimental overview of the strategy used to evaluate the effect of AGO2 depletion on leukemic development in two genetically distinct AML-PDX mouse models (AML-579 and AML-602; table S2). (**B**) Whole-body IVIS showing radiance signal at 10 weeks postinjection in mice injected with shAGO2 or shCTRL AML-PDX 579 cells. (**C**) Longitudinal IVIS radiance monitoring from weeks 2 to 12 posttransplantation in shAGO2 and shCTRL AML-PDX mice. Top: AML-PDX 579; bottom: AML-PDX 602. (**D**) Leukemic burden [log_2_(BLI) signal; week 10 versus week 2 posttransplantation] in AML-PDX 579 and AML-PDX 602 mice transduced with shCTRL, shAGO2.1, or shAGO2.2 (*n* = 4 per group). (**E**) Kaplan-Meier survival plots of mice transplanted with shAGO2 or shCTRL AML-PDX cells. Left: AML-PDX 579; right: AML-PDX 602. Statistical significance was assessed using the Mantel-Cox log-rank test between shAGO2 and shCTRL groups.

AGO2 depletion profoundly impaired leukemic development in both AML-PDX models. Whole-body IVIS imaging revealed markedly reduced radiance signals in AGO2-depleted mice compared with controls, which exhibited progressive leukemia expansion and elevated leukemic burden over time (Fig. 6, B and D). Consistent with these findings, bone marrow (BM) aspirates revealed a higher proportion of human AML blasts (HLA^+^/mCherry^+^) in control mice, a phenotype that was also detected in peripheral blood (PB) to a lesser extent (fig. S7B). Although AML-602 exhibited substantially slower disease kinetics and longer disease latency than AML-579, AGO2 depletion consistently impaired leukemic progression across both models and significantly prolonged survival (Fig. 6, B to E). Consistent with these observations, control animals displayed extensive leukemic infiltration of the BM, liver, and spleen at euthanasia in both AML-PDX models (fig. S7C). In the more aggressive AML-579 model, this was accompanied by marked splenomegaly (fig. S7D). In contrast, AGO2-depleted mice showed a profound reduction in tissue engraftment, with only occasional residual leukemic infiltration detected at the experimental end point (fig. S7C). As increased apoptosis could account for this phenotype, we assessed apoptosis in long-term ex vivo cocultures with MS5 stromal cells. AGO2 depletion did not result in a significant increase in apoptosis under these conditions (fig. S7, E to G). Combined with the impaired proliferative capacity and altered myeloid differentiation observed following AGO2 depletion (Fig. 4, J to M), these findings suggest that the reduced leukemogenic potential primarily reflects impaired leukemia maintenance rather than increased cell death.

Together, these findings establish AGO2 as a functional contributor to AML progression. The consistent impairment of leukemic development across independent AML-PDX models with distinct molecular characteristics demonstrates that AGO2 dependency extends beyond a specific genetic context and identifies AGO2 as a potential therapeutic vulnerability in AML.

## DISCUSSION

In this study, we applied an integrative multiomics framework combining DNAm, chromatin activity and conformation, transcriptional data, a newly generated TET2 binding map, and functional studies to delineate TET2-mediated regulatory programs during myeloid commitment. This approach revealed an enhancer-enriched subset of high-confidence TET2 targets, including a key regulatory element at the *AGO2* locus that undergoes lineage-specific demethylation and activation to regulate myeloid cell fate and function—epigenetic processes that become disrupted in patients with AML. Notably, AGO2 depletion impaired leukemia development in vivo, highlighting a functional axis with potential prognostic and therapeutic relevance.

By Flag-tagged pulldown in a physiological model, we reveal TET2 chromatin occupancy during human myeloid commitment, capturing a transitional stage not previously characterized in studies of fully differentiated myeloid cells (*20*). This approach showed that TET2 is recruited early during myeloid commitment, likely preceding widespread DNAm remodeling, highlighting its dynamic role in fate specification. Integration with complementary epigenomic and transcriptomic datasets revealed a pronounced TET2 bias toward regulatory elements—not only enhancers, as previously described (*37*), but also promoters of key myeloid genes, including *KLF4*, *ITGAM*, and *IL1B*—where binding correlated with stable or transient H3K27ac deposition and gene activation. Notably, we showed that TET2-bound promoters are largely unmethylated across the human hematopoietic system, making 5hmC an unreliable proxy for TET2 binding and underscoring the need for direct mapping to define TET2’s role in cell fate specification.

The presence of TET2 at unmethylated promoters suggests roles beyond demethylation during myeloid commitment. Notably, TET2 may shape chromatin architecture independently of DNAm, strengthening both local loops and long-range interactions (5 to 10 Mb) at its binding sites. This effect could involve biomolecular condensation of TET2 (*27*) and cooperative interactions with factors such as CEBPA (*28*). A previous study in the murine hematopoietic system has highlighted distinct biases induced by the loss of TET2 catalytic activity versus complete protein loss (*15*), supporting the idea that noncatalytic functions may have physiological relevance. However, the precise mechanisms of this noncatalytic remodeling and its functional implications in human myeloid commitment remain to be defined.

Conversely, we identified a discrete set of enhancer-enriched TET2-bound sites (T2-MDPs, methylation dynamic peaks) that acquired chromatin accessibility and active histone modifications concurrent with DNA demethylation during myeloid commitment, resulting in robust up-regulation of their associated genes. These genes are enriched for inflammatory and stimulus-responsive functions and may be regulated by the STAT1 and RELA/NFKB1 pathways. While the temporal order of demethylation and activation remains unresolved, our previous work demonstrated that CRISPR-dCas9–mediated DNA demethylation can trigger gene activation, as exemplified by *IL1RN* (*24*), a hallmark T2-MDP gene specifically demethylated in the myeloid lineage across the hematopoietic system. Complementing these findings, the functional interrogation of the *AGO2* regulatory regions further demonstrates that T2-MDPs can act as bona fide enhancers whose activation contributes directly to lineage-specific transcriptional programs and cell-fate control.

To further bridge our mechanistic findings with physiological relevance, we systematically validated our results in primary hematopoietic and AML datasets. Despite distinct cellular origins, we observed notable DNAm similarities at TET2 binding sites between in vitro and in vivo myeloid commitment. This analysis uncovered a previously unreported intragenic enhancer within the *AGO2* locus, where TET2-driven demethylation and *AGO2* activation dynamics closely tracked hematopoietic maturation toward the myeloid lineage. While previous studies have implicated AGO2 in granulocytic, monocytic, and erythroid differentiation (*31*, *32*, *35*, *38*, *39*), our data position AGO2 as a broader regulator of myeloid cell fate. AGO2 depletion accelerated acquisition of myeloid features, altered inflammatory responses, reduced proliferative capacity, and impaired leukemic propagation across independent AML-PDX models. Together, these findings suggest that AGO2 contributes to the balance between differentiation and self-renewal in both normal and malignant hematopoiesis. The identification of an AML-specific dependency on AGO2, together with the profound impairment of leukemic development observed across genetically distinct AML-PDX models, further supports a functional role for AGO2 in leukemia maintenance. However, the molecular events underlying these effects warrant further investigation, particularly given AGO2’s multifaceted regulatory potential—encompassing canonical RNA-induced silencing complex–dependent and noncanonical functions (*31*, *32*, *40*–*42*), as well as emerging roles in mRNA sequestration within P-bodies (*43*, *44*), a process recently linked to myeloid leukemia (*45*).

An intriguing finding from the AML analyses was that both *AGO2* hypermethylation and elevated *AGO2* expression were associated with adverse clinical outcome. Rather than reflecting a simple linear regulatory relationship, these observations suggest that *AGO2* methylation and *AGO2* expression may capture distinct biological features of AML. Notably, *AGO2* hypermethylation was strongly associated with AMLs carrying mutations in *TET2* and *IDH1/2*, linking disruption of the DNA demethylation machinery to aberrant regulation of this locus. In contrast, elevated *AGO2* expression identified transcriptionally distinct AML subgroups with erythroid and megakaryocytic features. These findings highlight the multifaceted contribution of the TET2-AGO2 axis to AML biology and suggest that epigenetic deregulation and AGO2-dependent transcriptional programs may influence disease progression through partially independent mechanisms.

Overall, our study uncovers a TET2-dependent regulatory mechanism at the *AGO2* locus, whereby lineage-specific enhancer demethylation fine-tunes *AGO2* expression to coordinate myeloid differentiation, inflammatory responsiveness, and proliferative capacity, while disruption of this regulatory axis may contribute to leukemic progression. Beyond mechanistic insight, these findings highlight AGO2 as a potential therapeutic target in AML, either through direct pharmacologic inhibition (*46*) or in combination with DNA-hypomethylating agents, offering a potential strategy to restrain leukemic growth while promoting differentiation-associated programs.

## MATERIALS AND METHODS

The research conducted in this study complies with all relevant ethical regulations. All experimental procedures were approved by the Animal Care Committee of the Barcelona Biomedical Research Park (DAAM 9667/DAAM11883).

### Mouse care and maintenance

Seven- to 16-week-old non-obese diabetic/NOD.Cg-Prkdcscid Il2rgtm1Wjl Tg(CMV-IL3,CSF2,KITLG)1Eav/MloySzJ (NSGS) mice (the Jackson Laboratory, Ban Harbor, ME; RRID: IMSR_JAX:013062), housed under pathogen-free conditions, were used as recipients for transplantation experiments. The animal rooms were fully air conditioned with a temperature of 20° to 24°C and 45 to 65% humidity, according to Annex A of the European Convention EC. The maximum stocking density of the cages corresponds to Annex III of the 2010/63 EU. The cages were constantly filled with structural enrichment, and the animals had unlimited access to food and water.

### Cell culture growth and maintenance

BLaER1 cells (BlaER) were derived from a human B cell precursor leukemia cell line (RCH-ACV) that stably expresses the myeloid TF CEBPA fused with the estrogen receptor and labeled with green fluorescent protein (*21*). BlaER cells were grown in suspension in RPMI 1640-Hepes (42401018, GIBCO) supplemented with 10% heat-inactivated fetal bovine serum (FBS; A5256701, GIBCO), 1% penicillin-streptomycin (15140122, GIBCO), 1% l-glutamine (25030024, GIBCO), and 550 μM β-mercaptoethanol (63689, GIBCO). The culture medium was replaced every 2 to 3 days, maintaining the cellular density between 0.2 × 10^6^ and 2 × 10^6^ cells per ml.

Human embryonic kidney (HEK) 293T cells were grown in Dulbecco’s modified Eagle’s medium (41965039, GIBCO) (+) d-glucose supplemented with 10% heat-inactivated FBS (A5256701, GIBCO), 1× penicillin-streptomycin (15140122, GIBCO), and 1× l-glutamine (25030024, GIBCO). AML-579 and AML-602 PDX cells (*47*, *48*) were cultured in RPMI 1640 (11875093, GIBCO) supplemented with 20% heat-inactivated FBS (A5256701, GIBCO), 5% l-glutamine (25030024, GIBCO), 1% gentamycin (15750060, GIBCO), 1% penicillin-streptomycin (15140122, GIBCO), 0.6% insulin-transferrin-selenium (41400045, Life Technologies), 1 mM sodium pyruvate (11360070, GIBCO), and 550 μM β-mercaptoethanol (63689, GIBCO). Clinical characteristics of AML-579 and AML-602 are listed in table S2.

MS5 stromal cells were grown in MEM-α GlutaMAX (32561037, GIBCO) supplemented with 10% heat-inactivated FBS (A5256701, GIBCO), 1× penicillin-streptomycin (15140122, GIBCO), and 2 mM sodium pyruvate (11360070, GIBCO). For optimal growth, all cell lines were kept in a 5% CO_2_ humidified atmosphere at 37°C. Cells were checked for mycoplasma infection every month and tested negative.

### In vitro myeloid commitment of human B cells into iMacs

To induce myeloid commitment of leukemic BlaER B cells into iMacs, cells were seeded at 0.2 × 10^6^ cells per ml in medium supplemented with 100 nM β-estradiol (E2) (E2758, Sigma-Aldrich) to activate CEBPA. In addition, human IL-3 (10 ng ml^−1^; 200-03, PeproTech) and macrophage colony-stimulating factor (10 ng ml^−1^; 300-25, PeproTech) were added to favor the conversion.

### Assessment of in vitro myeloid commitment by flow cytometry

Cells (0.1 × 10^6^) were blocked with human Fc receptor binding inhibitor (eBiosciences) for 10 min, followed by the addition of conjugated CD11b (CD11b-APC) and CD19 (CD19-PE). Antibody staining was performed in the dark for 20 min at room temperature. Before the acquisition, cells were washed once with phosphate-buffered saline (PBS) and stained with DAPI (4′,6-diamidino-2-phenylindole) for viability assessment. Sample acquisition was performed using the BD FACSCanto II Flow Cytometry System (BD Biosciences), and data analysis was carried out with FlowJo software. Fluorescence-activated cell sorting (FACS) antibody information and staining dilutions are listed in table S3.

### Genome editing vector construction

The TET2-3×FLAG-P2A-NeoR/HygR targeting vectors were cloned by serial modification of base vectors pMK292 (RRID: Addgene_72830), pMK293 (RRID: Addgene_72831), and pFETCh Donor (RRID: Addgene_63934). Briefly, 800-bp homology arms (5′ HA and 3′ HA) at the last coding exon of *TET2* were amplified from BlaER genomic DNA. The pMK backbone, NeoR/HygR, and 3×FLAG-P2A elements were amplified from pMK293, pMK292, and pFETCh donor plasmids, respectively. All fragments were assembled by Gibson reaction (*49*) for 1 hour at 50°C. Gibson assembly master mixes were prepared by the Protein Technologies Unit at the Centre for Genomic Regulation (CRG, Barcelona).

Guide RNAs (gRNAs) targeting *TET2* last coding exon, for 3×FLAG tagging, or exons 8 and 9, for *TET2*-KO, were cloned into the px330-mCherry (RRID: Addgene_98750) empty backbone. For *AGO2* ER1 and ER2 deletions, two pairs of gRNAs were designed to target each region. gRNAs located upstream and downstream of the ROIs were cloned into px330-mCherry (RRID: Addgene_98750) and px330-BFP (RRID: Addgene_64323), respectively. px330 cloning was performed following Zhang lab guidelines (www.addgene.org/static/cms/filer_public/04/43/04439a9b-fd21-419c-b872-426b7b0e122f/zhang-lab-general-cloning-protocol.docx). gRNA sequences are listed in table S4.

### shRNA knockdown vector construction

For Dox-inducible shRNA-mediated *TET2* silencing, oligonucleotide pairs encoding shRNAs were annealed and cloned into the Tet-pLKO-puro plasmid (RRID: Addgene_21915). For constitutive shRNA-mediated *AGO2* silencing, oligonucleotide pairs encoding shRNAs were annealed and cloned into pSICOR-PGK-puro (RRID: Addgene_12084) or modified pSICOR-BFP-puro (RRID: Addgene_31845) plasmids. shRNA sequences are listed in table S5.

### Generation and validation of genome-edited cell lines

To generate TET2-3×FLAG knocked-in cells, two rounds of transfection were performed with the TET2-3×FLAG-P2A-NeoR and TET2-3×FLAG-P2A-HygR plasmids to target both *TET2* alleles. Transfection was carried out by electroporation with Amaxa Nucleofector KitC (H3VCA-1004, Lonza), where 1 μg of TET2-3×FLAG-P2A-NeoR plasmid per 100 μl of Nucleofector solution mix was used to electroporate 1 × 10^6^ BlaER cells (Nucleofector II Device; program: X-001). Two days after transfection, neomycin at 500 μg ml^−1^ (N1142, Sigma-Aldrich) was added to the medium, which was changed every 2 to 3 days until cells recovered normal proliferation rates. Neomycin-resistant cells were then subjected to the same transfection procedures using the TET2-3×Flag-P2A-HygR plasmid to generate double-resistant cell lines selected with hygromycin at 100 μg ml^−1^ (ant-hg-1, Gibco). The bulk population resistant to the drug was sorted by FACS to generate single-cell clones. After 2 weeks of expansion, homozygous insertion was confirmed by genotyping polymerase chain reaction (PCR). To generate *TET2*-KO and *AGO2* ER1/2^−/−^ cells, transfections were performed as previously described. Two days after transfection, *TET2*-KO and *AGO2* ER1/2^−/−^ cells were single-cell sorted by FACS for mCherry^+^ and mCherry^+^/BFP^+^ populations, respectively, to generate clones. *AGO2* ER1/2^−/−^ clones were expanded for 2 weeks, and homozygous deletion was confirmed by genotyping PCR. *TET2*-KO clones were expanded for 4 weeks and validated by Sanger sequencing and TET2 Western blotting (see the “Western blots” section). Genotyping primers are listed in table S6.

### Cellular transduction for shRNA-mediated silencing

shTET2-Tet-pLKO-puro and shAGO2-pSicoR-puro BlaER cell lines and their respective controls were generated by lentiviral transduction. For each plasmid, low-passaged HEK-293T cells were seeded at 0.3 × 10^6^ to 0.5 × 10^6^ cells per ml and cotransfected with VSV-G and psPAX2 lentiviral plasmids using a calcium phosphate–mediated transfection protocol. Medium change was performed 12 hours posttransfection, and supernatants containing lentiviral particles were harvested and filtered (0.45 μm) at 48 hours posttransfection. Viral particles were concentrated at 70,000*g* for 2 hours at 10°C, and then the viral pellets were resuspended by agitation for 3 hours at 4°C. Then, 1 × 10^6^ BlaER cells were spin infected (1000*g*) at 32°C for 90 min, and 12 hours postinfection, cells were washed to remove debris and remaining viral particles.

Two days postinfection, puromycin at 1 μg ml^−1^ was added to the medium and maintained for 3 to 7 days to select the transduced cells. The selection medium was changed every 2 to 3 days.

For lentiviral transduction of AML-579 and AML-602 PDX cells, shAGO2-pSicoR-BFP or shCTRL-pSicoR-BFP viral supernatants were collected 24 and 48 hours after transfection and concentrated by centrifugation at 70,000*g* for 2 hours at 10°C. Concentrated virus was resuspended in PBS and stored at −80°C. Cells were transduced by spin infection with virus at 1000*g* for 90 min at 32°C in the presence of polybrene (8 μg ml^−1^).

### Xenotransplantation of primary human AML

For leukemic engraftment evaluation in AGO2-depleted conditions, AML-579 and AML-602 PDX-eFFly-mCherry cells were transduced with shAGO2-pSicoR-BFP and shCTRL-pSicoR-BFP (see the “Cellular transduction for shRNA-mediated silencing” section). Forty-eight hours posttransduction, cells were T cell depleted with an anti–CD3-OKT3 antibody (RRID: AB_3711423). To ensure the selection of transduced and viable AML-PDX, cells were then stained with APC-anti-human-HLA-ABC antibody and FACS sorted for HLA^+^/mCherry^+^ and BFP^+^ cells to reach a purity of more than 95%. For transplantation, sublethally irradiated (2 grays) sex- and age-matched NSGS mice (6 to 12 weeks old) received intratibial injections of 0.1 × 10^6^ PDX cells (*n* = 4 per group). FACS antibody information and staining dilutions are listed in table S3.

### AML PDX graft evaluation

shAGO2 and shCTRL PDX tumor burden was monitored biweekly by bioluminescence (BLI) (starting from 2 weeks postinjection) using Xenogen IVIS (PerkinElmer). Data were quantified with Aura Imaging Software. Mice were euthanized when disease symptoms were evident, and leukemia engraftment was incompatible with animal welfare (max. radiance >108 photons/s cm^−2^ sr^−1^) or body weight reduction of 20% or at the experimental end point (26 weeks postinjection).

At 10 weeks postinjection, PB was collected by facial vein bleeding and BM by aspiration from noninjected tibiae. The leukemic engraftment was analyzed by flow cytometry. To remove erythrocytes, PB and BM aspirates were lysed with BD FACS Lysing solution (349202, BD Biosciences) for 15 min. Next, to detect surface markers, cells were first incubated with anti-mouse CD16/CD32 Fc Block for 10 min to prevent background staining. Cells were then stained with an APC-anti-human-HLA-ABC antibody, protected from light for 20 min. Next, cells were washed and acquired using a BD LSRFortessa SORP (BD Biosciences). Human blasts were defined as HLA^+^/mCherry^+^ cells.

At the end point, all mice were analyzed for leukemic grafts in BM, liver, and spleen tissues, which were isolated after euthanasia and analyzed by flow cytometry, as previously described. FACS antibody information and staining dilutions are listed in table S3.

### AML-PDX in vitro coculture and apoptosis assays

MS5 stromal cells were inactivated by treatment with mitomycin C (M4287, Sigma-Aldrich) at a final concentration of 20 μg ml^−1^ for 3 hours at 37°C. Meanwhile, culture plates were coated with 0.1% gelatin and incubated at 37°C for 30 min. After treatment, cells were washed with PBS, trypsinized, and collected by centrifugation. The cell pellet was resuspended in fresh medium and plated onto gelatin-coated 24-well plates at a density of 3 × 10^4^ cells per well. Inactivated cells were used the following day for coculture experiments with AML-579 PDX cells. For coculture experiments, AML-PDX cells were cultured on MS5 stromal cells in StemSpan SFEM II (09655, STEMCELL Technologies) supplemented with CC100 (2690, STEMCELL Technologies) and 1× penicillin-streptomycin (15140122, GIBCO).

AML-PDX eFFly-mCherry cells were transduced with shAGO2-pSicoR-BFP and shCTRL-pSicoR-BFP (see the “Cellular transduction for shRNA-mediated silencing” section). Twenty-four hours posttransduction, cells were stained with an APC-anti human-HLA-ABC antibody, protected from light for 20 min. Next, cells were washed and FACS sorted to obtain HLA^+^ cells. Sorted cells were seeded over stromal cells at a density of 1 × 10^5^ cells per ml and kept in culture for 1 week.

To measure apoptosis, cells were incubated at room temperature and protected from light for 15 min with FITC (fluorescein isothiocyanate) anti-annexin V (640906, BioLegend) according to the manufacturer’s instructions. Sample acquisition was performed using the BD FACSymphony A1 Flow Cytometry System (BD Biosciences), and data analysis was performed with FlowJo software. FACS antibody information and staining dilutions are listed in table S3.

### *TET2* knockdown induction with Dox

To induce *TET2* knockdown in shTET2-Tet-pLKO cell lines, shRNA activity was triggered by the addition of Dox at 1 μg ml^−1^ (D989, Sigma-Aldrich) to the medium. Dox was added 48 hours before myeloid commitment induction and readded every 2 days during the process to maintain a sufficient concentration for shRNA expression at all time points. *TET2* reduction was confirmed by real-time quantitative PCR.

### Western blots

For protein extraction, up to 10 × 10^6^ cells were incubated in lysis buffer [50 mM tris-HCl (pH 7.5), 150 mM NaCl, 1 mM EDTA, 1 mM EGTA, 5 mM Mg_2_Cl, 0.5% Triton X-100, and proteinase inhibitors] on ice for 1 hour, followed by centrifugation at 12,000*g* for 15 min to remove cell debris. Up to 100 μg of clean protein extract was separated by electrophoresis in an 8% polyacrylamide gel and transferred to a nitrocellulose blotting membrane at 400 mA for 4 hours. Membranes were blocked with 5% nonfat milk in TBS-Tween (50 mM tris, 150 mM NaCl, and 0.1% Tween 20) for 30 min, with shaking at room temperature. Primary antibody incubation was done at 4°C overnight, followed by secondary antibody staining for 1 hour at room temperature, protected from light. The membranes were visualized using Odyssey CLx Imager (LI-COR). Antibody information and working concentrations are listed in table S7.

### Intracellular flow cytometry staining

For 5hmC and AGO2 intracellular staining, cells were fixed with 4% methanol-free formaldehyde for 15 min at room temperature following permeabilization and blocking with 0.1% Triton-X containing 2% donkey serum. For 5hmC staining, DNA was denatured with 2 M HCl for 30 min and neutralized with tris-HCl (pH 8.0) before the blocking step. Cells were incubated with the primary antibodies at room temperature for 2 hours for 5hmC or at 4°C for 1 hour for AGO2, followed by fluorochrome-conjugated secondary staining for 30 min, protected from light. AGO2 protein level evaluation in shAGO2 AML-579 cells was done 24 hours postinfection. Samples were acquired in the BD FACSymphony A1 Flow Cytometry System (BD Biosciences), and data analysis was performed with FlowJo software. Antibody information and working concentrations are listed in table S7.

### Proliferation assays

shAGO2 and shCTRL cells were plated at 0.2 × 10^6^ cells and monitored by flow cytometry for proliferation in either uninduced or myeloid conversion conditions with CellTrace Violet (C34570, Invitrogen). 7-Aminoactinomycin D was used as a viability dye. Manual counting was done in uninduced B cells at 5 days postseeding with a hemocytometer (trypan blue exclusion).

### Immunophenotypic analysis of myeloid surface markers

shAGO2 and shCTRL iMacs were collected and incubated for 10 min at room temperature with a human FcR binding inhibitor to avoid unspecific staining. Next, the cells were divided and single-antibody stained against the selected panel of myeloid surface markers for 20 min at room temperature in the dark. After a PBS wash and viability marker staining, cells were acquired in the BD FACSymphony A1 Flow Cytometry System (BD Biosciences), and data analysis was performed with FlowJo software. FACS antibodies and staining dilutions are listed in table S3.

### Cytokine measurements

The LEGENDplex Human M1/M2 Macrophage Panel (10-plex) (740508, BioLegend) was used to quantify cytokines in the supernatant of shAGO2 versus shCTRL iMacs. Cells were left untreated or stimulated for 24 hours with human TNFα (10 ng ml^−1^; 300-01A-50UG, PeproTech), LPS (200 ng ml^−1^; L2630-10MG, Sigma-Aldrich), or IL-1β (10 ng ml^−1^; 200-01B-10UG, PeproTech). Staining procedures were followed per the manufacturer’s protocol. Samples were acquired in the BD FACSymphony A1 Flow Cytometry System (BD Biosciences), and LEGENDplex Data Analysis Software (BioLegend) was used for data analysis.

### Real-time quantitative PCR

Total RNA was extracted using TRIzol (10296010, eBiosciences), and 0.5 μg of total RNA was converted into cDNA using the High-Capacity cDNA Reverse Transcription Kit (4368814, Applied Biosystems). Real-time quantitative PCRs were performed using SYBR Green Universal Master Mix (4309155, Applied Biosystems) and analyzed using the QuantStudio 5 System (Applied Biosystems). *HPRT1* was used as an internal control. Primer sequences are listed in table S8.

### TET2-FLAG ChIP-seq

TET2 binding sites were evaluated by FLAG-based immunoprecipitation in TET2-3×FLAG–tagged versus untagged control cells. Cells (50 × 10^6^) were collected at 72 hpi with two biological replicates per condition and washed twice in ice-cold PBS. Following a dual cross-linking ChIP protocol [modified from Broome *et al.* (*50*)], a fresh stock solution of 0.25 M disuccinimidyl glutarate (HY-114697, MedChemExpress) in dimethyl sulfoxide was prepared and diluted to 2 mM in PBS. Cell pellets were resuspended in 5 ml of disuccinimidyl glutarate cross-linking solution and incubated in rotation at room temperature for 30 min followed by two PBS washes. Next, the samples were dual cross-linked with 1% formaldehyde (F8775, Sigma-Aldrich) in rotation for an additional 10 min at room temperature. To quench the reaction, 0.125 M glycine was added, and samples were incubated in rotation for 5 min at room temperature.

Cross-linked cells were washed twice in ice-cold PBS and resuspended in cold immunoprecipitation buffer {1 volume of SDS buffer [100 mM NaCl, 50 mM tris-HCl (pH 8.1), 5 mM EDTA (pH 8), 0.2% NaN_3_, and 0.5% SDS]:0.5 volume of Triton dilution buffer [100 mM NaCl, 100 mM tris-HCl (pH 8.6), 5 mM EDTA (pH 8), 0.2% NaN_3_, and 5% Triton X-100] supplemented with proteinase inhibitors (Roche)}. Chromatin was sheared to 100- to 300-bp fragments using a Bioruptor Pico Sonicator (Diagenode) at 4°C for 13 cycles (30 s on/30 s off) in 15-ml Bioruptor Pico Tubes (C30010017, Diagenode). The sonicated chromatin was spun down at 20,000*g* for 20 min at 4°C to remove any insoluble portion, and the supernatant was precleared with 20 μl of Dynabead A/G mix (10003D and 10001D, Invitrogen). All chromatin was incubated with 5 μg of monoclonal anti-FLAG M2 antibody (F3165, Sigma-Aldrich; RRID: AB_259529) overnight at 4°C in rotation. The next day, 50 μl of Dynabead A/G mix (1:1) was blocked with bovine serum albumin (5 mg ml^−1^) and added to the sample for immunoprecipitation for 4 hours at 4°C in rotation.

Chromatin-antibody-bead complexes were washed three times with ice-cold low-salt buffer [50 mM Hepes (pH 7.5), 140 mM NaCl, and 1% Triton X-100] and once with ice-cold high-salt buffer [50 mM Hepes (pH 7.5), 500 mM NaCl, and 1% Triton X-100]. After the last wash, the complexes were decrosslinked in elution buffer (1% SDS and 0.1 M NaHCO_3_) by overnight incubation at 65°C with shaking at 1300 rpm. The next day, the eluted portion was treated with ribonuclease A (20 ng ml^−1^) for 1 hour at 37°C followed by proteinase K (200 ng ml^−1^) for 2 hours at 65°C. The immunoprecipitated DNA was purified by phenol:chloroform:isoamyl alcohol (25:24:1) extraction.

For chromatin immunoprecipitation sequencing (ChIP-seq), samples were subjected to quality control and quantification with Agilent 2100. Library preparation and sequencing were performed by an external sequencing service provider using a DNABSEQ-G400 (MGI Tech) sequencer.

### RNA-seq

shTET2 (2 × 10^6^ cells) and shAGO2 (2 × 10^6^ cells) with their respective shCTRL cells were collected at 0 (B cell) and 72 and 144 (iMac) hours post–myeloid induction, with at least two biological replicates per group. RNA was extracted with the RNeasy Mini Kit (74104, QIAGEN) and subjected to quality control with Agilent 2100 to ensure high RNA integrity (RIN > 7). For library preparation, ribosomal RNA removal was done either with poly-A enrichment (shTET2 RNA-seq) or ribonuclease H (shAGO2 RNA-seq) following the DNABSEQ Eukaryotic Strand-specific mRNA pipeline. Library preparation and sequencing were performed by the external sequencing service provider using a DNABSEQ-G400 (MGI Tech) sequencer.

### DNAm arrays

shTET2 and shCTRL cells (1 × 10^6^) were collected at 0 (B cell) and 72 and 144 (iMac) hours post–myeloid induction, with three biological replicates per group. DNA was extracted with the Wizard Genomic DNA Purification Kit (A1120, Promega), and 1 μg of genomic DNA was bisulfite converted using the EZ DNA Methylation Gold Kit (D5006, Zymo Research). One hundred to 500 ng of bisulfite-converted DNA was hybridized to Infinium MethylationEPIC version 1.0 Bead-Chip arrays. Hybridization procedures were performed by the Genomics Unit of the Josep Carreras Leukaemia Research Institute following the manufacturer’s instructions.

### DNAm validation by pyrosequencing

Bisulfite-converted DNA (see the “DNAm arrays” section) from shTET2 and *TET2*-KO iMacs, together with their respective controls, was used for pyrosequencing validation of DNA methylation levels at the *AGO2* ROIs (ER1 and ER2). Converted DNA was PCR amplified using the IMMOLASE DNA Polymerase Kit (BIO-21046, Bioline), and the resulting amplicons were sequenced using the PyroMark Q48 system (QIAGEN). Primer design and DNAm analysis were performed using PyroMark Assay Design 2.0 software (QIAGEN). Primer sequences are listed in table S9.

### Bioinformatic analyses

All new sequencing and array data obtained were mapped onto the human genome assembly hg38 (Ensembl GRCh38). All bioinformatic analyses were done with Python (version 3.11.9) and R environments (4.2.1) using packages from the Bioconductor suite (version 3.0) (*51*). Genomic heatmaps, average plots, and bigwig tracks were generated using deepTools (version 3.3.1) (*52*). The integration of different datasets was performed using the bedtools package (version 2.31.1) (*53*). GO enrichment analyses were performed using clusterProfiler (version 4.4.4) (*54*). Custom plots integrating GO enrichment with expression data were done with the plotGODESeq package (https://gitlab.com/ngamblen/plotGODESeq). Gene set enrichment analysis (GSEA) was done using the fgsea package (version 1.32.2) (*55*). Ternary diagrams were generated with the ggtern package (3.5.0). The MA plots were generated using the ggmaplot function from the ggpubr R package (version 0.6.0). Heatmaps and clustering analyses were performed using the ComplexHeatmap (version 2.10.0) (*56*) and deepTools computeMatrix (version 3.3.1) tools (*52*). PCA was performed using limma (version 3.52.2) (*57*) and the prcomp function from the stats package (version 4.4.0). UpSet plots were generated using the upsetR package (version 1.4.0) (*58*). Correlations were calculated with the sm_statCorr from the smplot2 R package (version 0.2.5). Kaplan-Meier survival analysis plots were done with the survit function from the survival package (version 3.8-3) and plotted with the survminer package (version 0.5.0). The remaining plots were generated using the R package ggplot2 (v3.4.2).

### TET2 ChIP-seq analysis

Reads were trimmed using the TrimGalore package (version 0.6.6) to remove adaptors and mapped using Bowtie2 (version 2.4.4.1) (*59*). Duplicated reads were removed with the tool MarkDuplicates from the package Picard (version 3.1.0) (Picard Tools, https://broadinstitute.github.io/picard). Peaks were called against the control samples using MACS2 (version 2.2.5) (*60*), with parameter (-q 0.05). For peak calling, regions overlapping the “Encode blacklist” regions were removed (*61*), as well as mitochondrial reads. Peaks were annotated to genomic features in R with the package ChIPseeker (version 1.32.1) (*62*). Biological replicates were handled with the IDR method (*63*) using the package idr (version 1.3).

### Hi-C analysis

Raw Hi-C interactions at 0 (B cell) and 72 and 144 (iMac) hpi were processed with the HiC-Pro (version 3.1.0) (*64*) pipeline following the recommended parameters with ICE normalization. Normalized contact maps at a 5-kb resolution were used for downstream analysis.

The matrices were normalized and corrected with the hicNormalize function to the lower sequencing depth (--smallest) and hicCorrectMatrix function, respectively, from the HiCExplorer package (version 3.7.6) (*65*). Publicly available Hi-C datasets from healthy hematopoiesis were converted from HiC “.pairs” format to 5-kb binned cool files with the cooler package (version 0.10.3) and normalized as previously described. The contacts heatmap was plotted with the HiContacts package (version 3.21) (*66*).

### Identification of TET2 bound regulatory regions

For the CRE definition, intersection of ATAC-seq (assay for transposase-accessible chromatin with high-throughput sequencing) peaks with H3K4me1 and TSSs was done as previously described (*23*). The evaluation of H3K27ac signal was done with the Diffbind package using the DBA_DESEQ2 method (version 2.2.12). H3K27ac loss or gain was defined by comparing iMac versus B cell time points [signal (FC) > 2; false discovery rate (FDR) <0.05]. H3K27ac transient gain was defined by an increase in signal at 72 hpi versus B cell and iMac stages [signal (FC) > 1; FDR < 0.05]. The H3K27ac transient loss group accounted for only 1.05% (*n* = 42) of all regions; hence, it was excluded for clarity in the downstream analyses. TET2 binding sites were overlapped with the defined CREs using the foverlaps function from the data.table package (version 1.17.6)

### TET2 E-P loop definition

Hi-C data integration with TET2 site CRE classification (see the “Identification of TET2-bound gene regulatory regions” section) was used to define E-P sites. Contact intensity between bin pairs where TET2 overlapping occurred on both sites was extracted from “bedpe” files and classified according to their CRE definition (E-P, E-E, and P-P). Only the bin pairs where the E-P loop occurred were used for the downstream analysis. E-P bin pairs not bound by TET2 were randomized and used as non–TET2-bound control. Gene expression integration considered only the promoter-associated gene for the quantification.

### Long-range interactions

Aggregate peak analysis (APA) showing long-range interactions between TET2 binding sites was performed using the HiCExplorer package (version 3.7.6) tool hicAggregateContacts. Hi-C matrices were generated at a 5-kb resolution using HiCExplorer and long-range interactions (5 to 10 Mb) between the provided coordinates. APA quantification was done following ENCODE guidelines for Hi-C processing and analysis (www.encodeproject.org/documents/75926e4b-77aa-4959-8ca7-87efcba39d79/@@download/attachment/comp_doc_7july2018_final.pdf).

### WGBS analysis and quantification

WBGS data during myeloid commitment were processed as previously described (*24*). DNAm quantification within TET2 binding sites was calculated from normalized bigWig files with the Python numpy.nanmean function (version 2.1). Only confident CpGs (>3× coverage) were considered, and changes in DNAm equal to or greater than 15% were classified as dynamic.

Methylome data from 30 AML samples with distinct mutational profiles were obtained from previous studies [*n* = 18 (*67*) and *n* = 12 (*68*)]. Preprocessed bigWig files were used for the downstream analysis. For DNAm quantification at the *AGO2* locus, only IDR < 0.05 TET2 binding coordinates were considered as ROIs (R1 to R7). Coordinate lift-over from hg19 to hg38 [from Spencer *et al.* (*68*)] was done with the UCSC Lift Genome Annotations tool (https://genome.ucsc.edu/cgi-bin/hgLiftOver). Sample IDs are listed in table S10.

### RNA-seq analysis

For shTET2 and shAGO2 RNA-seq datasets, reads were mapped using STAR with standard options (version 2.7.6) (*69*). Gene expression was quantified using the function featureCounts from the package Subread (version 2.0.3) (*70*). DEGs (FDR < 0.05) were detected using the R package DESeq2 (version 1.36.0) (*71*). DESeq2 or vsd count normalization was used for further analysis, unless stated otherwise

Normalized expression for healthy primary hematopoiesis from the Blueprint consortium (http://blueprint-data.bsc.es/#!/about) was obtained from preprocessed RNA-seq data using the RSEM gene-level estimated counts using the R package DESeq2 (version 1.36.0) following the tximport() pipeline.

To obtain DEGs from patients with AML, raw counts from the BeatAML2 (https://biodev.github.io/BeatAML2/) and TCGA-LAML cohorts were processed as previously described. BeatAML2 patients’ overall survival, leukemia classification, and mutational status were extracted from the Clinical Summary report (https://biodev.github.io/BeatAML2/).

### TF motif enrichment analysis

TET2 site motif enrichment analysis was performed using MEME Suite, a motif-based sequence analysis tool. The Simple Enrichment Analysis (version 5.5.7) (*72*) tool was used to determine relative TF enrichment with shuffled input sequences as the background (https://meme-suite.org/meme/tools/sea).

### TF activity inference

The TFs’ activity estimation (activation or inhibition) was done with decoupleR (version 2.14.0) (*73*) using the CollecTRI regulons (*74*). Significant *t* values (stat) from DEGs (iMac versus B cell) detected in T2-MSPs and T2-MDPs groups were used for the analysis.

### Cancer dependency analysis

AGO2 cancer dependency was obtained from the Dependency Map (DepMap) portal (https://depmap.org/portal/). CRISPR (DepMap Public 26Q1 + Score, Chronos) and Expression Public 26Q1 datasets were used for the analyses.

### DNAm array analysis

IDAT raw data from the Infinium MethylationEPIC version 1.0 Bead-Chip arrays were processed in R following the minfi pipeline (version 1.42.0) (*75*). Probes with a low detection *P* value (<0.01), probes with a known single-nucleotide polymorphism at the CpG site, and known cross-reactive probes were removed. For the resulting CpGs, β-values were calculated using minfi functions. DMPs were calculated using the limma package (version 3.52.2), adjusting by the Benjamini-Hochberg method. Only DMPs with an absolute Δβ-value >0.15 and *P* < 0.01 were considered for the downstream analysis.

Publicly available Infinium HumanMethylation450 BeadChip IDAT raw data from the TCGA-LAML cohort were processed as previously described. For differential analysis in Fig. 5B, the patient sample cohort was selected, excluding carriers of other mutations that might affect DNAm, such as *DNMT3A*, *IDH1/2*, *WT1*, *and CEBPA* (*6*, *29*, *76*–*78*). Patients’ overall survival and mutational status was extracted from the cBioPortal database (www.cbioportal.org/) (*79*, *80*). Sample IDs are listed in table S11.

### Statistics and reproducibility

Details for statistical analyses, including the number of biological replicates and/or sample size, are provided in the figure legends. Statistics were calculated using the R stats package (version 4.4.0). Data normality was tested before conducting statistical analyses.

In figures, all box plots display the first and third quartiles as the lower and upper edges of the box, with a line inside the box showing the median value and whiskers extending to 1.5 times the interquartile range. All bar plots show the means ± SD. An unpaired two-tailed Student’s *t* test was used to determine statistical significance in a two-sided manner, unless otherwise specified. Error bars represent standard deviation. When comparing gene expression, “differential gene expression” statistics are shown (see the “RNA-seq” section). Statistical significance for Kaplan-Meier survival curves was calculated using the Mantel-Cox log-rank test, and *P* values for linear regression fit were calculated using two-sided Pearson correlation, unless otherwise specified.
