# Supplementary material for: TET2-driven activation of AGO2 links epigenetic remodeling to myeloid commitment and leukemia
Source: Sci Adv. 2026 Oct 23;12(43):eaee5701. doi: 10.1126/sciadv.aee5701 (PMC13693683; doi:10.1126/sciadv.aee5701)
Supplement: Supplementary file 1 [file sciadv.aee5701_sm.pdf]

Science Advances
AAAS

Supplementary Materials for

TET2-driven activation of AGO2 links epigenetic remodeling to myeloid commitment and leukemia

Aleksey Lazarenkov *et al.*

Corresponding author: José Luis Sardina, jsardina@carrerasresearch.org

Sci. Adv. **12**, eaee5701 (2026)
DOI: 10.1126/sciadv.aee5701

The PDF file includes:

Figs. S1 to S7
Tables S1 to S9
Legends for tables S10 and S11

Other Supplementary Material for this manuscript includes the following:

Tables S10 and S11

SUPPLEMENTARY FIGURES

Lazarenkov_Supplementary_Figure_1

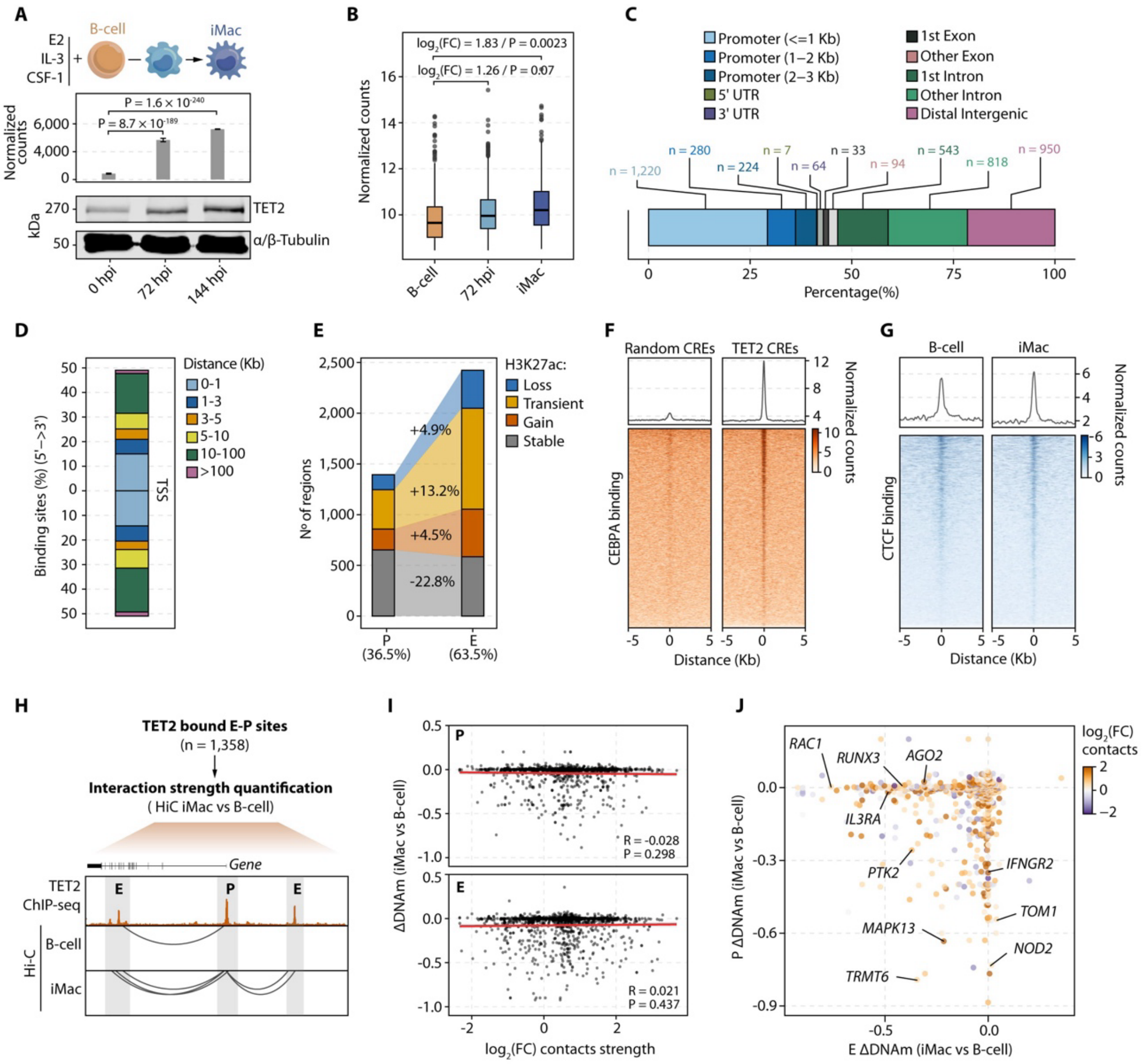

fig. S1. Related to Fig.1. (A) Top: Schematic overview of the cellular model used to study myeloid commitment. Middle and bottom: TET2 mRNA and protein levels during myeloid commitment. **(B)** Boxplots displaying levels of highly expressed macrophage genes detected to be significantly changing during myeloid commitment (iMac vs B-cell). Data from BioGPS Macrophage Gene Set (Harmonizome 3.0) (n = 929). **(C)** Genomic features of TET2 binding sites. **(D)** Distribution of TET2 binding sites relative to the nearest Transcription Start Site (TSS). **(E)** Stacked barplots showing chromatin activity dynamics at TET2-bound promoters and enhancers during myeloid commitment, based on H3K27ac changes. Percentage change is normalized to the total number of regions in each group. **(F)** Average plot (Top) and genomic heatmap (Bottom) showing CEBPA occupancy at TET2-bound CREs compared with random CREs in iMac. **(G)** Average plot (Top) and genomic heatmap (Bottom) of CTCF occupancy at TET2-bound CREs in B-cells and iMacs. **(H)** Schematic overview of the strategy used to define TET2-bound enhancer-promoter (E-P) pairs. **(I)** Scatterplots showing the correlation between DNAm

and contact strength at promoters (P) and enhancers (E) during myeloid commitment (iMacs vs B-cell). The solid line represents the linear regression fit, and the shaded area denotes the 95% confidence intervals. P values were calculated from a two-sided Pearson correlation. **(J)** Scatterplot depicting changes in DNAm and contact strength at TET2-bound E-P pairs during myeloid commitment (iMac vs B-cell).

Lazarenkov_Supplementary_Figure_2

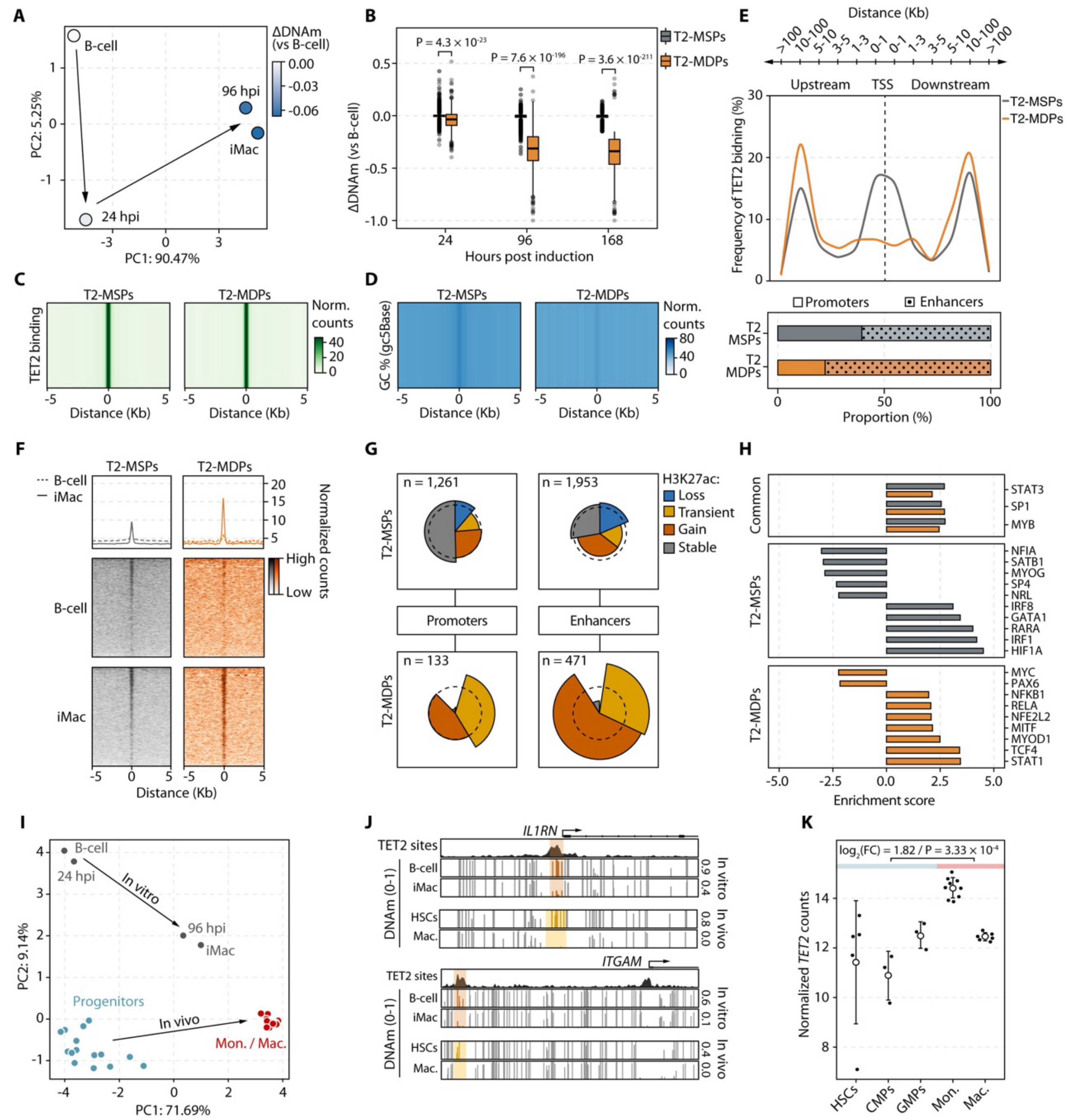

Fig. S2. Related to Fig. 2. (A) Principal Component Analysis (PCA) of the DNAm dynamics at TET2 binding sites during myeloid commitment. Arrows indicate inferred DNAm trajectories. Color indicates the average DNAm changes during myeloid commitment (relative to B-cells). **(B)** Boxplots showing DNAm changes (relative to B-cells) during myeloid commitment at T2-MSPs/MDPs. **(C)** Genomic heatmap showing TET2 ChIP-seq signal intensity at T2-MSPs/MDPs. **(D)** Genomic heatmap showing the CG content enrichment at T2-MSPs/MDPs. **(E)** Top: Distribution of T2-MSPs/MDPs relative to the nearest Transcription Start Site (TSS). Bottom: Proportion of the associated CREs at the T2-MSPs/MDPs. **(F)** Average plots (Top) and genomic heatmaps (Bottom) of CEBPA ChIP-seq signal at T2-MSPs/MDPs. **(G)** Combined pie chart and radar plot showing relative enrichment of TET2-bound CREs and their activation kinetics (H3K27ac levels) within T2-MSP/MDPs. The radius of each pie

segment represents the fold enrichment of T2-MSP/MDPs dynamics relative to all CREs, with the dashed line indicating an enrichment value of 1. **(H)** Transcription Factor (TF) activity enrichment inferred from expression changes of genes associated with T2-MSPs/MDPs during myeloid commitment (iMac vs B-cell). Top 10 most enriched (positively or negatively) TF activities are shown. **(I)** Principal Component Analysis (PCA) of the DNAm dynamics at T2-MDPs during “in vitro” myeloid commitment model (B-cell to iMac) vs. primary “in vivo” (HSCs to macrophages) differentiation. Arrows indicate inferred DNAm trajectories. Progenitors: HSCs: Hematopoietic Stem Cells (n = 9), CMPs: Common Myeloid Progenitors (n = 3), GMPs: Granulocyte-Monocyte progenitors (n = 3). Mon.: Monocytes (n = 9); Mac.: macrophages (n = 6). **(J)** Genome browser snapshot showing example T2-MDPs shared between the *in vitro* and *in vivo* myeloid commitment systems at the *IL1RN* and *ITGAM* loci. Shaded regions highlight TET2 binding sites. Average DNAm levels within each region are shown on the right. **(K)** *TET2* expression (RNA-seq) dynamics during primary myeloid commitment. Differential expression analysis was assessed between committed (Mon.;Mac.) and progenitor (HSCs, CMPs, GMPs) cells. Progenitors: HSCs: Hematopoietic Stem Cells (n = 5), CMPs: Common Myeloid Progenitors (n = 3), GMPs: Granulocyte-Monocyte progenitors (n = 3). Mon.: Monocytes (n = 9); Mac.: macrophages (n = 6).

Lazarenkov_Supplementary_Figure_3

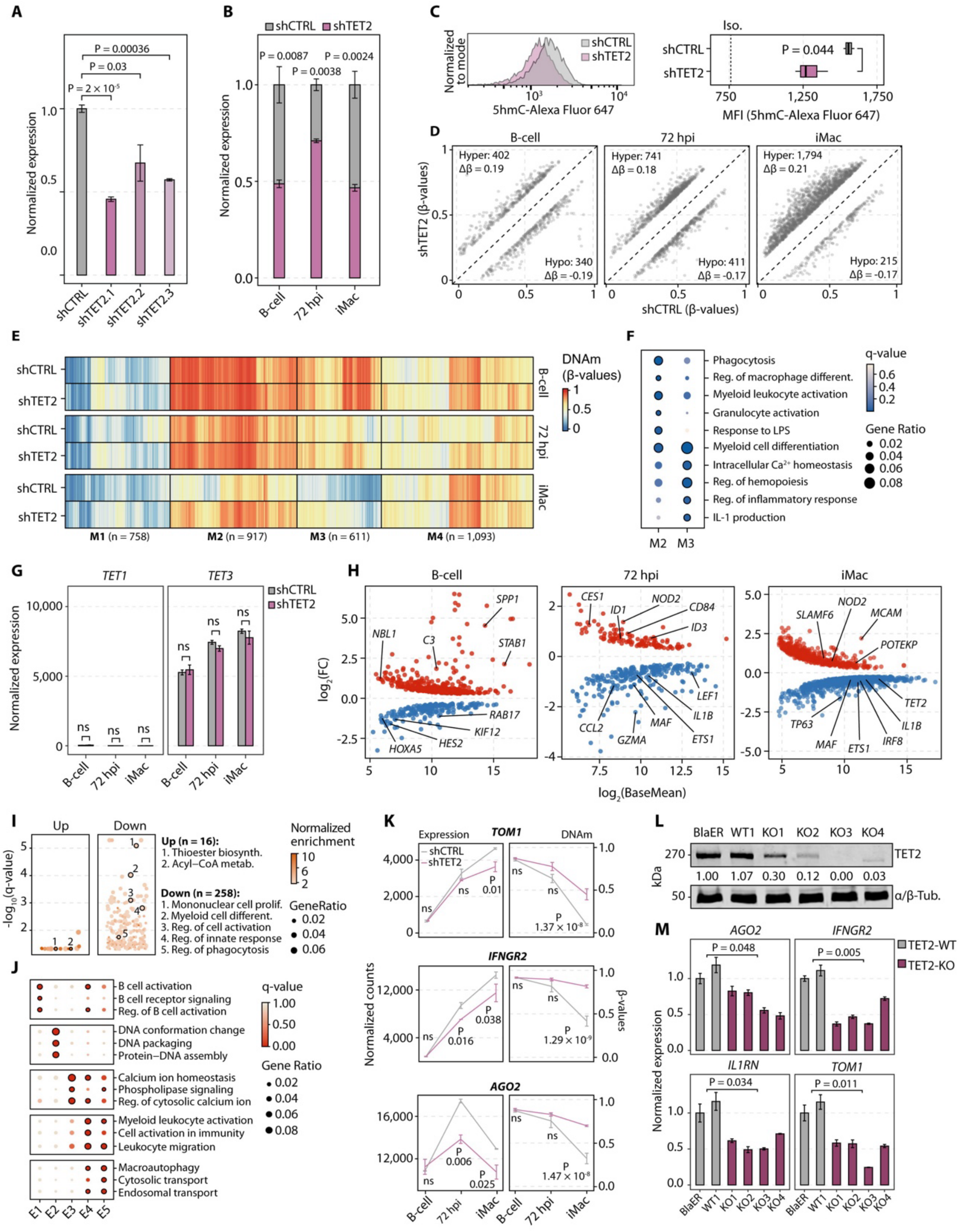

fig. S3. Related to Fig. 3. (A) *TET2* expression (RT-qPCR) in B-cells transduced with different TET2-targeting shRNAs. Values were normalized to the housekeeping gene (HPRT) and TET2 levels in shCTRL cells (n = 3). **(B)** Barplots showing *TET2* expression (by RT-qPCR) in shTET2 (shTET2.1) and control cells during myeloid commitment. Values were normalized as in (A) at each timepoint (n = 3). Endogenous TET2 expression peaks at 72 h during myeloid commitment (Fig. S1A). **(C)** 5hmC levels (by Intracellular Flow Cytometry) in shTET2 B-cells. Top: representative histograms of 5hmC

signal intensity. Bottom: quantification of signal (n = 3 biological replicates). MFI: Mean Fluorescence Intensity. Iso.: Isotype Control. **(D)** Scatterplots showing Differentially Methylated Positions (DMPs) detected in shTET2 vs. shCTRL cells during myeloid commitment. **(E)** Heatmap showing the clustering of DMPs (M1-M4) detected in shTET2 versus control cells during myeloid commitment. **(F)** Gene Ontology (GO) enrichment analysis of genes associated with M2- and M3-DMP clusters. Selected biological processes from the top 20 most significant terms are shown. Terms with q-value < 0.05 are outlined in black. **(G)** TET1 and TET3 expression (RNA-seq) in shTET2 vs shCTRL conditions during myeloid commitment (n = 2). **(H)** MA plots of Differentially Expressed Genes (DEGs) detected in shTET2 cells during myeloid commitment. **(I)** GO enrichment analysis of the significant up- or downregulated DEGs detected in shTET2 iMacs. Selected biological processes are highlighted with a black outline. **(J)** GO enrichment analysis for the genes associated with expression clusters (E1-E5) from **Fig. 3F**. Selected biological processes from the top 10 most significant terms are shown; significant terms (q-value < 0.05) are outlined in black. **(K)** Expression and DNAm (β-values) levels of representative genes from DEGs/DMPs overlap in shTET2 versus control cells during myeloid commitment. **(L)** TET2 protein levels and its quantification in TET2-KO cells. Values were normalized to α/β-Tubulin and expressed as a fold change relative to WT cells**.** **(M)** Expression of representative genes (RT-qPCR) in TET2-KO iMac. Values were normalized to the housekeeping gene (HPRT) and WT cells (n = 3).

Lazarenkov_Supplementary_Figure_4

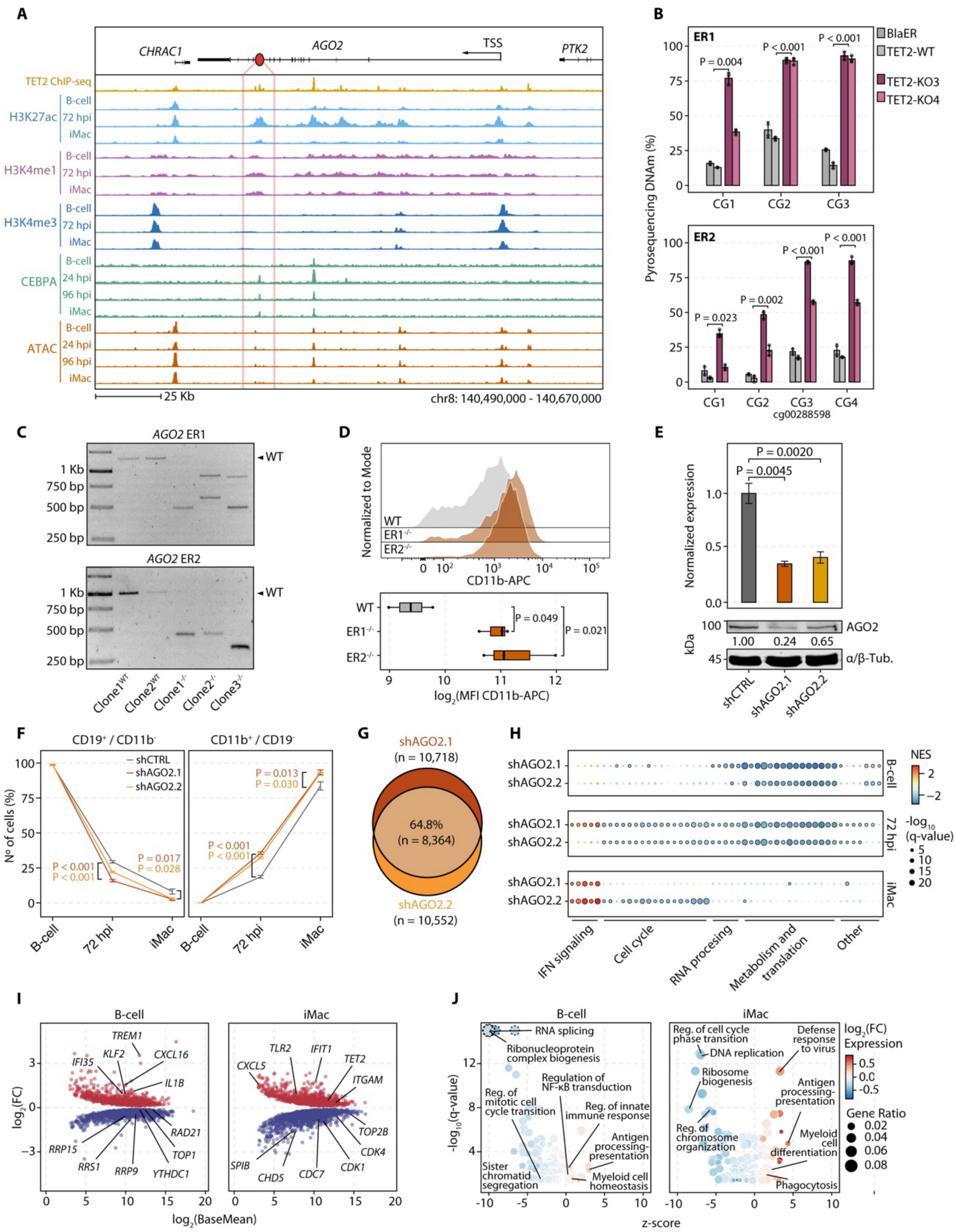

fig. S4. Related to Fig. 4. (A) Genome browser snapshot showing chromatin dynamics and CEBPA-binding at the *AGO2* locus during myeloid commitment. The region of interest ER1/2 identified in **Fig. 4** is shown as a red dot. **(B)** ER1/2 DNAm pyrosequencing validation in TET2-KO vs. WT cells (n = 3). **(C)** PCR validation of deleted regions in ER1$^{-/-}$ and ER2$^{-/-}$ clones. **(D)** CD11b surface levels (FACS) in ER1$^{-/-}$ and ER2$^{-/-}$ iMacs. Top: representative histograms of CD11b signal intensity. Bottom:

quantification of CD11b signal. MFI: Mean Fluorescence Intensity. **(E)** Evaluation of *AGO2* knockdown efficiency. Top: *AGO2* expression (RT-qPCR) in B-cells transduced with different AGO2-targeting shRNAs. Values were normalized to the housekeeping gene (HPRT) and *AGO2* levels in shCTRL cells (n = 3). Bottom: Corresponding AGO2 protein levels normalized to α/β-Tubulin and expressed as a fold change relative to shCTRL. (**F**) FACS analysis of cell surface markers in shAGO2 vs shCTRL cells. Percentages of B-cells (CD19+/CD11b-) and iMac (CD11b+/CD19-) are shown (n = 3). **(G)** Venn-diagram showing the overlap of all DEGs detected in the two AGO2-targeting shRNAs. **(H)** Gene Set Enrichment Analysis (GSEA) of DEGs in AGO2-targeting shRNAs cells during myeloid commitment. Commonly enriched terms from 'REACTOME', 'HALLMARK' and 'GOBP' gene sets are shown and grouped based on their biological function. NES: Normalized Enrichment Score. **(I)** MA plots showing DEGs in shAGO2 conditions at B-cell and iMac stages. **(J)** Gene Ontology (GO) enrichment analysis of DEGs in shAGO2 cells at B-cell and iMac stages. Over- and under-represented (z-score) biological processes are plotted, with color indicating the average log2(FC) of genes within each term. Outlier terms ($-\log_{10}$(q-value) > 15) are indicated by a dotted line.

Lazarenkov_Supplementary_Figure_5

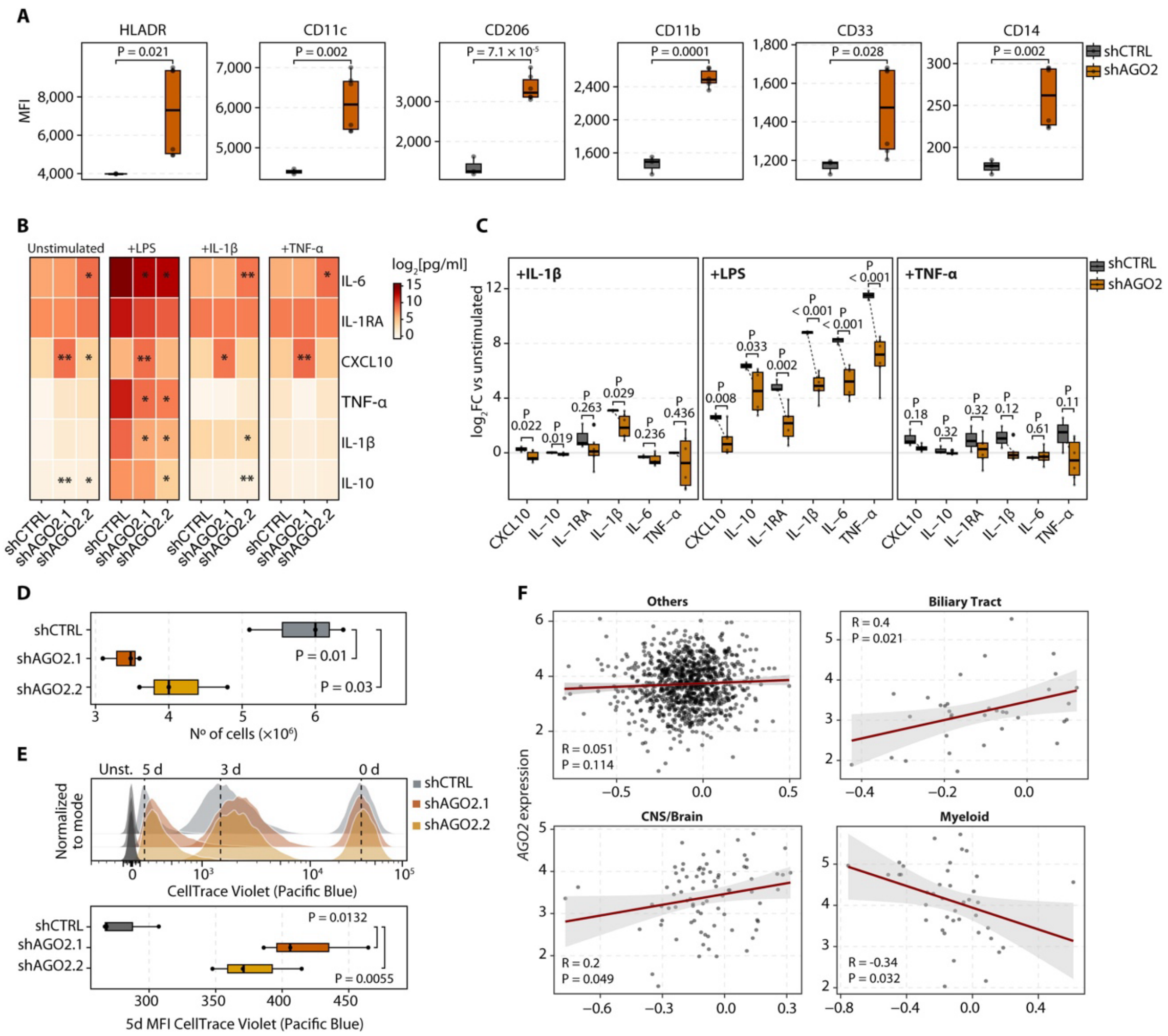

fig. S5. Related to Fig. 4. (A) Quantification of selected myeloid surface markers (FACS) in shAGO2 vs. shCTRL iMacs. **(B)** Heatmap of the cytokines concentrations in response to 24 hours treatment with different stimuli in shAGO2 vs shCTRL iMacs. (*P < 0.05; **P < 0.01; ***P < 0.001; ****P < 0.0001). **(C)** Quantification of cytokine release normalized to unstimulated levels in shAGO2 vs shCTRL iMacs in response to 24 hours treatment with different stimuli. LPS: Lipopolysaccharide. **(D)** Trypan Blue Exclusion assay showing cell counts of shAGO2 versus control uninduced B-cells at 5 days post-seeding (n = 3 biological replicates). **(E)** Proliferation assay by flow cytometry in shAGO2 versus control uninduced B-cells. Top: representative histograms showing CellTrace Violet signal. Bottom: quantification of CellTrace Violet signal at 5 days post-seeding (n = 3). MFI: Mean Fluorescence Intensity. Unst: Unstained control. **(F)** Scatterplots showing the correlation between *AGO2* expression and gene dependency in cell lines of different cancer types. Data from DepMap portal (https://depmap.org/portal/). The solid line represents the linear regression fit, and the shaded area denotes the 95% confidence intervals.

Lazarenkov_Supplementary_Figure_6

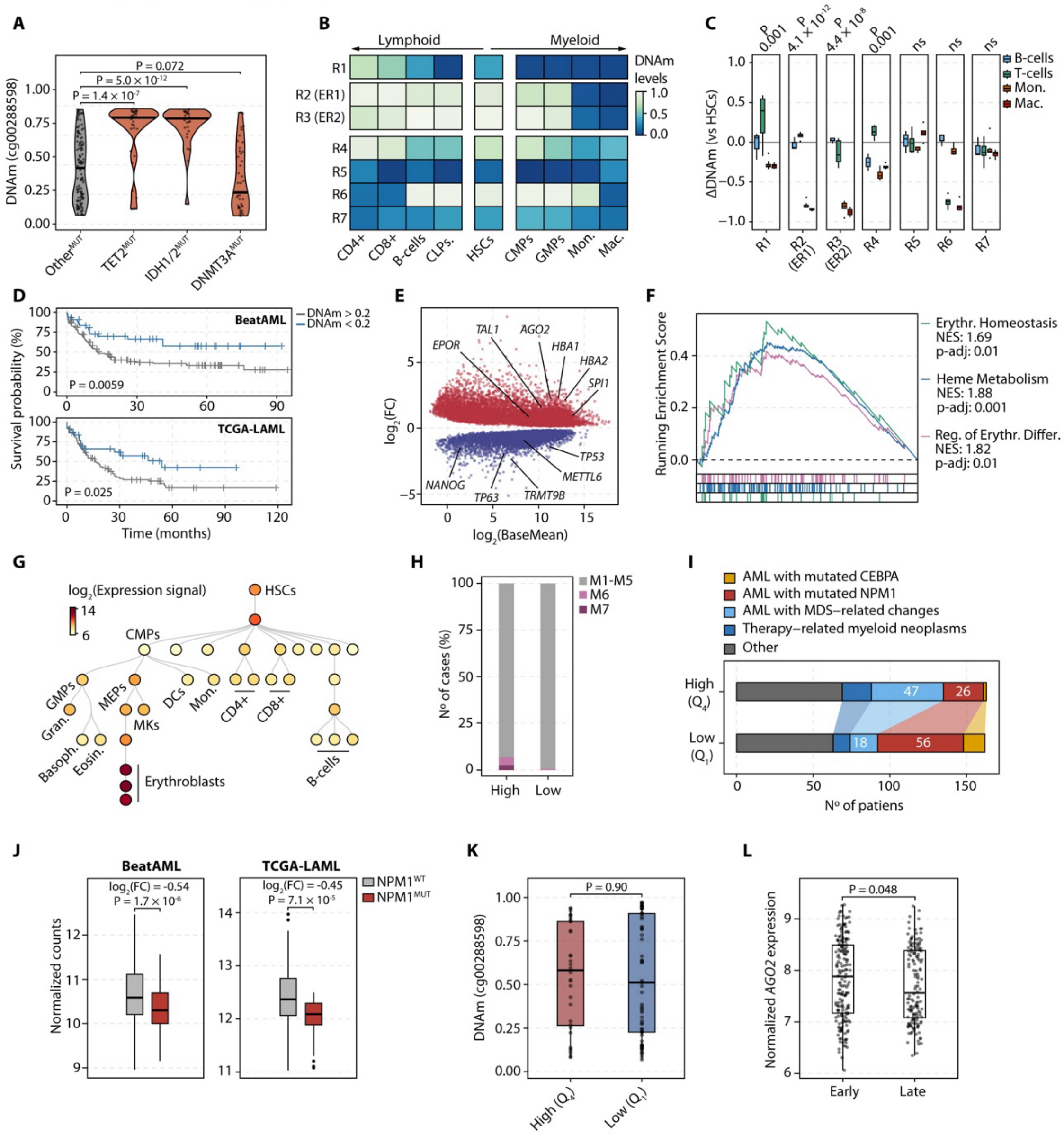

Fig. S6. Related to Fig. 5. (A) DNAm quantification of the AGO2 cg00288598 probe in patients with different DNAm altering mutations (data from BEAT-AML). **(B)** Heatmap showing DNAm levels at *AGO2* ROIs (R1-R7) during healthy hematopoiesis. **(C)** Boxplots showing the *AGO2* DNAm quantification at ROIs (R1-7) in mature hematopoietic populations. Statistical comparisons were performed between lymphoid (B-cells, CD4+, CD8+) and myeloid (Mon., Mac.) linages. n: HSCs = 9, CLPs = 3, CD8+ = 3, CD4+ = 3, B-cells = 3, CMPs = 3, GMPs = 3, Mon. = 9, Mac. = 6. Mon.; monocytes, Mac.: macrophages. **(D)** Kaplan-Meier survival curves of AML patients from the TCGA-LAML and BEAT-AML cohorts stratified by *AGO2* cg00288598 DNAm levels. **(E)** MA plot showing DEGs between patients with high (Q_4: Quartile 4; n = 163) versus low (Q_1: Quartile 1; n = 162) *AGO2* expression from the BEAT-AML2 cohort. **(F)** Gene Set Enrichment Analysis (GSEA) of the DEGs in high (Q_4) vs. low (Q_1) AGO2 patients from

the BEAT-AML2 cohort. Selected erythroid-related terms with significant enrichment are plotted. NES: Normalized Enrichment Score. **(G)** Hematopoiesis tree displaying *AGO2* expression across lineages (data from BloodSpot 3.0). **(H)** Stacked barplots showing the proportion of M6 (Acute erythroid leukemia) and M7 (Acute megakaryocytic leukemia) French-American-British (FAB) classified AML subtypes in AGO2 high (n = 165) versus low (n = 165) expressing patients (BEAT-AML2 cohort). “Other” includes M0-M5, NOS. **(I)** Stacked barplots showing AML subtype distribution at diagnosis in AGO2 high (Q_4) versus low (Q_1) expressing patients (BEAT-AML2 cohort). **(J)** Boxplots showing *AGO2* expression in NPM1-mutated versus wild-type AML samples from BEAT- AML2 (left) and TCGA-LAML (right) cohorts. **(K)** cg00288598 DNAm levels in patients with High (Q_4) and Low (Q_1) *AGO2* levels. **(L)** *AGO2* expression in Early vs Late DNAm loss groups.

Lazarenkov_Supplementary_Figure_7

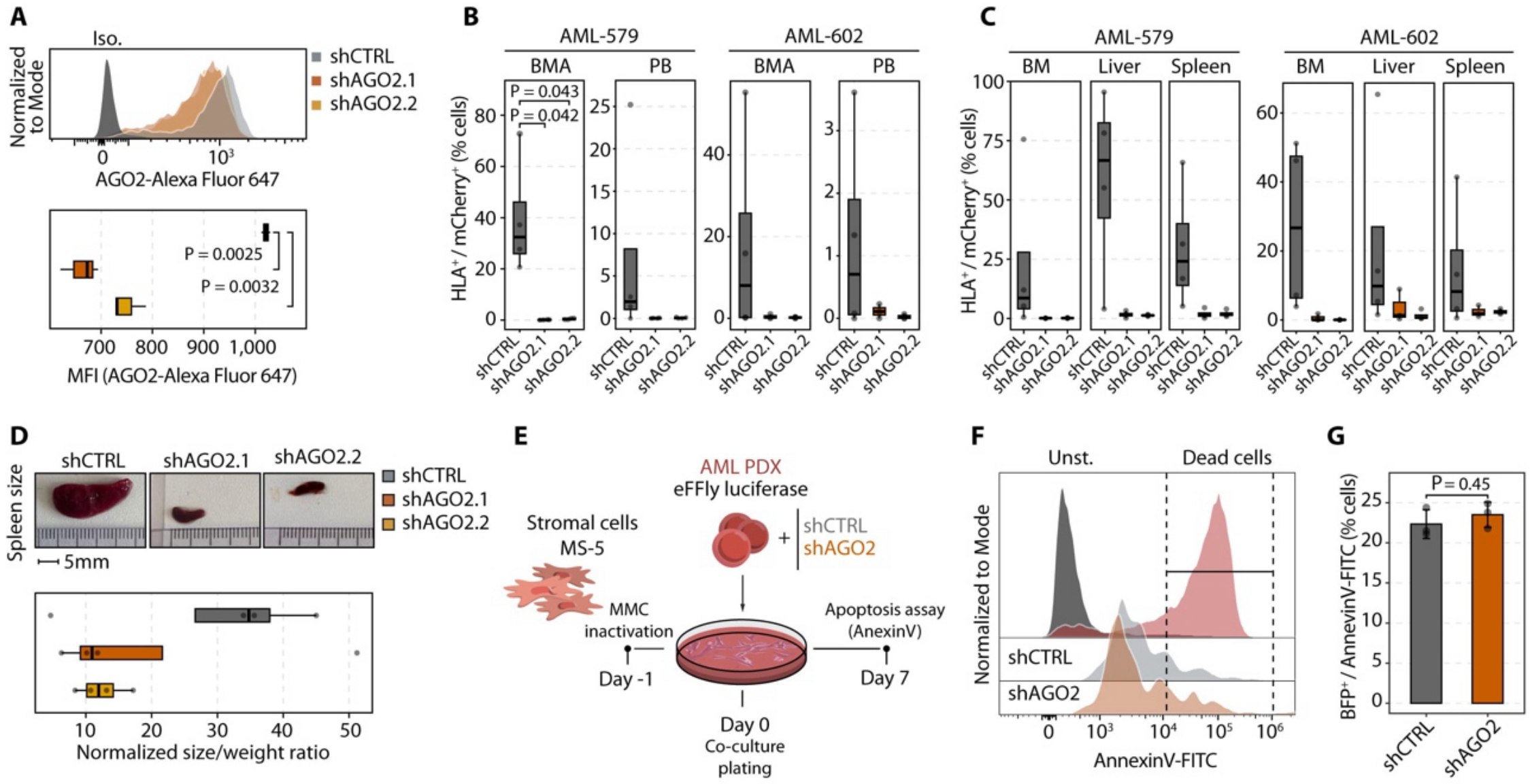

Fig. S7. Related to Fig. 6. (A) AGO2 protein levels (Intracellular Flow Cytometry) in shAGO2 AML-579 cells one day after infection. Top: Representative histograms showing AGO2 signal intensity. Bottom: Quantification of AGO2 signal (n = 3). MFI: Mean Fluorescence Intensity. Iso.: Isotype Control. **(B)** Leukemic engraftment quantification (FACS) in bone marrow aspirates (BMA) and peripheral blood (PB) from mice carrying shAGO2 or shCTRL AML-PDX cells. **(C)** Leukemic engraftment quantification (FACS) in organs of mice engrafted with shAGO2 or shCTRL AML-PDX cells at the experimental endpoint. BM: Bone Marrow. **(D)** Spleen size evaluation in AML-579 PDX mice. Top: Representative photos of spleens at euthanasia. Bottom: Boxplots showing the spleen's weight/size ratio normalized to the animal's body weight. **(E)** Schematic overview of the strategy used to assess apoptosis in shAGO2 vs shCTRL AML-579 *in vitro*. MMC: Mitomycin C. **(F)** Representative histograms showing AnnexinV signal (FITC) in shAGO2 vs shCTRL AML-579 after 7 days of *in vitro* culturing. Unst: Unstained control. **(G)** Quantification of AnnexinV signal (FITC) in shAGO2 vs shCTRL AML-579 after 7 days of *in vitro* culturing.

SUPPLEMENTARY TABLES

Supplementary Table 1. List of high-confident catalytic TET2 targets identified in **Fig. 4A.**

Gene Name	chr	start	end	DNAm B-cell	DNm 24hpi	DNm 96hpi	DNAm iMac
HDAC9	chr7	18639083	18639368	0.93	0.91	0.00	0.00
FKBP15	chr9	113187940	113188657	0.95	0.96	0.30	0.21
SNX11	chr17	48113245	48113605	0.89	0.93	0.40	0.24
NDRG1	chr8	133245659	133246391	0.89	0.83	0.36	0.26
LHFPL2	chr5	78529463	78529952	0.80	0.82	0.24	0.19
NAGK	chr2	71065207	71065597	0.58	0.58	0.15	0.02
TOM1	chr22	35301485	35301923	0.76	0.66	0.29	0.22
PCBP1-AS1	chr2	70043440	70043841	0.83	0.65	0.27	0.30
SH3BP5L	chr1	248816967	248817394	0.73	0.56	0.33	0.20
PLB1	chr2	28463990	28464259	0.59	0.36	0.10	0.15
TMCC3	chr12	94675999	94676419	0.55	0.55	0.34	0.13
STXBP2	chr19	7656974	7657404	0.88	0.88	0.45	0.52
IFNGR2	chr21	33402220	33402848	0.77	0.77	0.49	0.42
FGL1	chr8	17907704	17908155	0.40	0.33	0.10	0.06
AGO2	chr8	140543587	140543977	0.65	0.67	0.31	0.34
NPL	chr1	182795658	182795905	0.29	0.22	0.09	0.07

Supplemental Table 2. Clinical characteristics of AML Patients used for PDX in vivo experiments in **Fig. 6.**

Sample	Age (years)	Sex	Disease stage	Cytogenetics	Mutations
AML-579	51	M	1*st* relapse	Normal	DNMT3A R882C, DNMT3A F868L, NPM1 L287fs, FLT3-ITD, IDH1 R132H
AML-602	40	F	1*st* relapse	Complex	DNMT3A, NPM1 W288Cfs*12, FLT3-ITD, TET2 N281*, TET2 S1369*, CEBPA D262Rfs*59, JAK3 V722I

Supplemental Table 3. FACS antibodies.

Usage	Antibody	Fluorophore	Brand	Catalog#	Clone	RRIDs	uL/test
Cell surface markers	CD19	PE	BD Biosciences	555413	HIB19	AB_395813	2.5
	CD11b	APC	BD Biosciences (BD OptiBuild)	550019	ICRF44	AB_398456	2.5
	HLA-DR (HLADR)	APC-FIRE 750	BioLegend	307657	L243	AB_2572100	2.5
	CD206	Alexa Fluor 647	Biolegend	321116	1502	AB_571881	2.5

	CD14	APC	BD Biosciences	561383	M5E2	AB_10612009	2.5
	CD11c	BUV661	BD Biosciences (BD OptiBuild)	612968	B-ly6	AB_2916887	2.5
	CD33 (Siglec-3)	PE	BD Biosciences	555450	WM53	AB_395843	2.5
	Anti-human-HLA-ABC	APC	BD Biosciences	555555	G46-2.6	AB_398603	20
Viability markers	Annexin V	FITC	BioLegend	640906	NA	AB_2561292	2.5
	DAPI	NA	Sigma-Aldrich	D9542	NA	NA	1:1000
	7AAD	NA	Thermofisher Scientific	A1310	NA	NA	1:100
Blocking	Anti-Hu Fc Receptor Binding	NA	eBioscience	16-9161-73	Polyclonal	AB_469272	1:100
	Anti-mouse CD16/CD32	NA	BD Biosciences	553141	2.4G2	AB_394656	1:500

Supplemental Table 4. gRNAs sequences used for CRISPR/Cas9 editing experiments.

Name	Sequence	Target
sgT2.Flag	ATGATATCACCCCCTTTTGT	TET2-tagging
sgT2.1	CATGCAGAATGGCAGCACAT	TET2-KO
sgT2.2	TTGGTGCCATAAGAGTGGAC	TET2-KO
sg1_ER1	TGTAGTTCCTGCTACCTAGG	ER1 deletion
sg1.1_ER1	GTATATGCGTGGGAGTGGTG	ER1 deletion
sg2_ER1	GGGAGGTCTGAAGGGCAAGG	ER1 deletion
sg2.1_ER1	TGGGAGAGAGGTGTCTGGAG	ER1 deletion
sg1_ER2	CGGTGCACTCTCAACCCGGG	ER2 deletion
sg1.1_ER2	ACCCTGAGTATATCCCAGTG	ER2 deletion
sg2_ER2	AGAGCTCATCGGTGACCTTG	ER2 deletion
sg2.1_ER2	AGTGGACGGTGGGAGCCAGG	ER2 deletion

Supplemental Table 5. shRNA sequences used for KD experiments.

Name	Sequence	Target
shCTRL	GTGGACTCTTGAAAGTACTAT	None
shTET2.1	TTTCACGCCAAGTCGTTATTT	TET2
shTET2.2	CAGTCTAATGTACGAACTTTA	TET2
shTET2.3	CAGATGCACAGGCCAATTAAG	TET2
shCTRL	GTGGACTCTTGAAAGTACTAT	None
shAGO2.1	GCAGGACAAAGATGTATTA	AGO2
shAGO2.2	GCAGGACAAAGATGTATTA	AGO2

Supplemental Table 6. PCR primers used for genotyping genome-edited clones.

Name	Sequence	Target
T2.Flag_F	CATGAAACTTCAGAGCCCACTTAC	TET2-tagging
T2.Flag_R	AGATAACCTCTTTTGTTGCTGGTG	TET2-tagging
ER1.del_F	CCCATCAGAGGAAAGGGCTG	ER1 deletion
ER1.del_R	CTCACTAAGCTGCGGTAGGG	ER1 deletion
ER2.del_F	GGCTCACCCTTCTACCCTCT	ER2 deletion
ER2.del_R	CAGCGTCTGCAGGTCATTTG	ER2 deletion

Supplemental Table 7. Western blot and intracellular staining antibodies.

Antibody	Dilution	Brand	Catalog#	Clone	RRIDs
rabbit anti-human TET2	1:1000	Abcam	Ab94580	Polyclonal	AB_10887588
rabbit anti-human AGO2 (for WB)	1:1000	Cell Signaling	2897	C34C6	AB_2096291
mouse anti-human α/β-Tubulin	1:5000	Cell Signaling	2148	Polyclonal	AB_2288042
goat anti-rabbit Alexa Fluor 680	1:10000	Thermo Fisher Scientific	A21109	Polyclonal secondary	AB_2535758
goat anti-rabbit Alexa Fluor 790	1:10000	Thermo Fisher Scientific	A11375	Polyclonal secondary	AB_2534146
rabbit anti-human 5hmC	1:500	Active Motif	39769	Polyclonal secondary	AB_10013602
mouse anti-human AGO2 (for IFC)	1:400	Proteintech	67934-1-Ig	1G2H12	AB_2918686
goat anti-mouse Alexa Fluor 647	1:400	Thermo Fisher Scientific	A21235	Polyclonal secondary	AB_2535804
goat anti-rabbit Alexa Fluor 647	1:400	Thermo Fisher Scientific	A21245	Polyclonal secundary	AB_2535813
TET2 monoclonal antibody	1:1000	Diagenode	C15200179	C15200179	AB_3344036
monoclonal anti-FLAG M2 antibody	1:1000	Sigma Aldrich	F3165	M2	AB_259529

Supplemental Table 8. RT-qPCR primers.

Name	Sequence	Target
HPRT_F	GACCAGTCAACAGGGGACAT	*HPRT*
HPRT_R	CTGCATTGTTTTGCCAGTGT	*HPRT*
TET2 _F	TACCGAGACGCTGAGGAAAT	*TET2*
TET2_R	ACATGCTCCATGAACAACCA	*TET2*
AGO2_F	CAAGTCGGACAGGAGCAGAAAC	*AGO2*
AGO2_R	GACCTAGCAGTCGCTCTGATCA	*AGO2*
IFNGR2_F	CTCCATTCTGCCTGGGTGACAA	*IFNGR2*

IFNGR2_R	CGTGGAGGTATCAGCGATGTCA	*IFNGR2*
TOM1_F	GAACTTCCACGAGGTGATGCTG	*TOM1*
TOM1_R	ATGGTCCTCACCAGCACACTCT	*TOM1*
IL1RN_F	TGCAAGCCTTCAGAATCTGGG	*IL1RN*
IL1RN_R	TCCTTGCAAGTATCCAGCAACT	*IL1RN*

Supplemental Table 9. Primers sequences for pyrosequencing.

Name	Sequence	Target	Use
ER1.pyro_F	TGTGTTAGGTATAGTGTTAGGGGTTTA	ER1	Amplification
ER1.pyro_R	AATATAATAACCCCTATACCCCCACAACAT	ER1	Amplification
ER2.pyro_F	TGTTTGTTGGAAATTAGGTATATGAG	ER2	Amplification
ER2.pyro_R	AACCTTAAAAAAAAAAACCCTATTACTAT	ER2	Amplification
ER1.seq1	GTAAGAGAAATAAGAAAATAGAAG	ER1	Sequencing
ER1.seq2	AGTAAGGGTTTGTTTGGG	ER1	Sequencing
ER1.seq3	GGGTTAGGGGTTGTG	ER1	Sequencing
ER1.seq4	AGGTATAGTGTTAGGGGTTTAA	ER1	Sequencing
ER2.seq1	ACTAAATCCAAACTCTACC	ER2	Sequencing
ER2.seq2	AAAAACCAACCCAAACAATAAT	ER2	Sequencing

Supplemental Table 10. AML patients' WGBS data used for *AGO2* DNA methylation stratification. Table provided in the accompanying Supplementary Excel file.

Supplemental Table 11. Samples from TCGA-LAML cohort used to identify TET2^{MUT} specific differentially methylated positions (Infinium HumanMethylation450 BeadChip). Table provided in the accompanying Supplementary Excel file.
